# Flagellar stator genes control a trophic shift from obligate to facultative predation and biofilm formation in a bacterial predator

**DOI:** 10.1128/mbio.00715-24

**Published:** 2024-07-22

**Authors:** Abhirup Mookherjee, Mohor Mitra, Gal Sason, Polpass Arul Jose, Maria Martinenko, Shmuel Pietrokovski, Edouard Jurkevitch

**Affiliations:** 1 Department of Plant Pathology and Microbiology, Faculty of Agriculture, Food and Environment, The Hebrew University of Jerusalem, Rehovot, Israel; 2 Department of Molecular Genetics, Weizmann Institute of Science, Rehovot, Israel; University of Hawaii at Manoa, Honolulu, Hawaii, USA; University of Puget Sound, Tacoma, Washington, USA

**Keywords:** *Bdellovibrios*, stator, predation, flagellar motility, cyclic di-GMP

## Abstract

The bacterial predator *Bdellovibrio bacteriovorus* is considered to be obligatorily prey (host)-dependent (H-D), and thus unable to form biofilms. However, spontaneous host-independent (H-I) variants grow axenically and can form robust biofilms. A screen of 350 H-I mutants revealed that single mutations in stator genes *fliL* or *motA* were sufficient to generate flagellar motility-defective H-I strains able to adhere to surfaces but unable to develop biofilms. The variants showed large transcriptional shifts in genes related to flagella, prey-invasion, and cyclic-di-GMP (CdG), as well as large changes in CdG cellular concentration relative to the H-D parent. The introduction of the parental *fliL* allele resulted in a full reversion to the H-D phenotype, but we propose that specific interactions between stator proteins prevented functional complementation by *fliL* paralogs. In contrast, specific mutations in a pilus-associated protein (Bd0108) mutant background were necessary for biofilm formation, including secretion of extracellular DNA (eDNA), proteins, and polysaccharides matrix components. Remarkably, *fliL* disruption strongly reduced biofilm development. All H-I variants grew similarly without prey, showed a strain-specific reduction in predatory ability in prey suspensions, but maintained similar high efficiency in prey biofilms. Population-wide allele sequencing suggested additional routes to host independence. Thus, stator and invasion pole-dependent signaling control the H-D and the H-I biofilm-forming phenotypes, with single mutations overriding prey requirements, and enabling shifts from obligate to facultative predation, with potential consequences on community dynamics. Our findings on the facility and variety of changes leading to facultative predation also challenge the concept of *Bdellovibrio* and like organisms being obligate predators.

**IMPORTANCE:**

The ability of bacteria to form biofilms is a central research theme in biology, medicine, and the environment. We show that cultures of the obligate (host-dependent) “solitary” predatory bacterium *Bdellovibrio bacteriovorus*, which cannot replicate without prey, can use various genetic routes to spontaneously yield host-independent (H-I) variants that grow axenically (as a single species, in the absence of prey) and exhibit various surface attachment phenotypes, including biofilm formation. These routes include single mutations in flagellar stator genes that affect biofilm formation, provoke motor instability and large motility defects, and disrupt cyclic-di-GMP intracellular signaling. H-I strains also exhibit reduced predatory efficiency in suspension but high efficiency in prey biofilms. These changes override the requirements for prey, enabling a shift from obligate to facultative predation, with potential consequences on community dynamics.

## INTRODUCTION

Biofilms are composed of microorganisms adhering to a surface and embedded in a self-produced matrix of extracellular polymeric substances (EPSs) (1, 2). They enhance cell protection from environmental stressors, support increased community diversity and interactions, and are a quantitatively prominent microbial way of life (2). Within biofilms, predatory interactions between protists, phages, predatory bacteria, and prey affect community diversity, food webs, evolution, virulence, and more (3–6). Among them, the predatory bacteria *Bdellovibrio* and like organisms (BALOs) efficiently consume prey in suspension and in biofilms which they can disperse (7–10) and shape microbial community dynamics, including infections by pathogens (5, 11–16).

BALOs are small bacteria, considered as obligate predators (host-dependent, H-D) of gram-negative bacteria (17). However, host-independent (H-I), facultative predators that grow on rich media in the absence of prey cells are readily isolated from H-D strains (18, 19), and at least one H-I strain was directly isolated from nature (20).

BALOs’ life cycle includes a highly motile prey search attack phase (AP) and a prey-dependent growth and replication phase (GP), which in most BALOs occur in the prey’s periplasm, as with *Bdellovibrio bacteriovorus*, the best studied BALO (21). At the end of GP, progeny AP cells exit the dead prey cell (22). Obligate H-D strains are therefore unable to form self (axenic—as a single species, without prey) biofilm, in contrast to H-I variants (23), which conserve their ability to prey, and are thus facultative predators (24). H-I mutants also exhibit AP-like and GP-like cell morphologies in rich medium (Fig. S1) and are deregulated in gene and protein expression (25, 26). Yet the genetics behind the H-D to H-I transition and the latter’s ability to build biofilms are poorly understood.

Here, we demonstrate that single mutations in stator genes are sufficient to bring about a shift from obligate H-D to facultative predation and robust H-I growth, to increase surface attachment, reduce motility, inhibit biofilm formation, change motility and cyclic-di-GMP-associated (CdG) gene expression, and alter CdG cellular concentration. In contrast, robust H-I growth and genuine biofilm formation require enabling mutations in poorly growing strains mutated in a protein associated with invasion pole pili (Bd0108) (19, 27, 28). We propose that mutations in stator genes by provoking motor instability, or alterations in the Bd108 invasion pole pili-associated protein, disrupt intracellular CdG signaling. This overrides the requirements for prey cues (29), enabling a shift from obligate to facultative predation, with potential consequences on community dynamics.

## MATERIALS AND METHODS

### Isolation of H-I strains

A freshly grown H-D100Sm culture with 10^8^ cells/mL was twice filter-sterilized with a 0.45 µm syringe filter to remove prey cells. One milliliter of this filtrate was centrifuged at 13,000 rpm for 10 min. Type I H-I mutants requiring prey extract for growth were obtained by spreading the cell pellet resuspended in 100 µL amHEPES over amPYE agar plates (amended media [am], with 3 mM MgCl_2_·6H_2_O and 2 mM CaCl_2_·2H_2_O) further amended with 7% (vol/vol) of an autoclaved *Escherichia coli* cell extract and streptomycin (Sm; 50 µg/ml) (30), while Type II H-I strains, which grow on rich medium, were selected on Sm PYE agar plates without prey extract (28). Plates were incubated at 28°C for H-I colony growth. Each colony was directly inoculated in 200 µL microtiter wells (Nunc MicroWell 96-Well plate, Thermo-Fisher) filled with amPYE medium with or without prey extract at 28°C under static conditions for 3 days. Thereafter, 10 µL of each culture was re-inoculated and incubated in the adequate fresh amPYE medium for subsequent biofilm-formation screening. Validation that the H-I isolates originated from H-D *B. bacteriovorus* was obtained by PCR amplification of the *bd0108* gene with 3F and 913R primer set (Table S1). Growth curves of selected H-I strains in PYE were prepared by continuous monitoring of the change in optical density at 600 nm (OD_600_) in microtiter wells, in a plate reader, at 28°C (Tecan, Switzerland).

### H-I biofilm growth

Biofilm formation in polystyrene microwell plates was measured using crystal violet (CV) (23, 31). Wells were thrice washed with sterile distilled water to remove planktonic cells. A CV solution (200 µL, 1%) was added to each well and incubated for 20 minutes, then removed, and the wells were washed with autoclaved water 3–4 times. Plates were air-dried, and 200 µL of a 33% acetic acid solution was pipetted into each well. Following 20 minutes of incubation, the wells were mixed by pipetting, and 150 µL was transferred to a fresh microtiter well. CV absorption was measured at OD_600_. Final CV adsorption values represent the mean of four replicate wells subtracted from values of the PYE medium without cells. The XTT [2,3-bis(2-methoxy-4-nitro-5-sulfophenyl)−2Htetrazolium-5-carboxanilide] reduction assay was used to evaluate the viability of the bacterial cells within the biofilms following (32). Two hundred microliter of XTT reagent solution was added to wells containing washed biofilms as above, and the plates were incubated for 2 h at 37°C. Thereafter, the solutions were mixed by pipetting, and the supernatants were measured at OD_490_.

H-I biofilms were also grown in microfluidics devices. A biofilm former (BFF) strain expressing the *tdtomato* (*tdt*) gene from plasmid pMQ414 (32) was inoculated into a six-channel μ-Slide VI 0.4 (ibidi) filled with amPYE medium, and incubated for 1 day, enabling initial biofilm development. Next, amPYE medium containing antibiotics (50 µg/mL of each streptomycin and gentamycin) was fed into the system at a low flow rate of 150 µL/h for 7 days to prevent contamination and to sustain the *tdt*-carrying pMQ414 plasmid.

### Gliding and swimming motility

Plaque diameter, a parameter that can reflect gliding or flagellar motility, was measured at different concentrations of top agar medium, in double-layered agar plates. Swimming motility was tracked to account for flagellar motility, including swimming speed and swimming patterns. Speed was measured in amHEPES and at different concentrations of polyvynylpyrolydonne (PVP; molecular weight 360,000, Sigma-Aldrich) to increase viscosity (33). For this, AP cells were obtained by co-culturing predator isolates along with *E. coli* ML35 prey for 24 hours and filtered to remove remaining prey cells. AP cells were added to amHEPES buffer at various viscosities to a final cell concentration of about 10^7^ cells/mL. Five microliter of the solution was put onto a glass slide (26 mm × 76 mm, Objektträger, Knittel glass, Germany) previously pre-warmed to remove moisture that may affect measurements. Slides were immediately examined under a 100× objective using an inverted microscope (Nikon TiE, Japan) at 22°C. Ten or more 10-second videos were captured without delay using a CMOS monochrome camera (DS-Qi2, Nikon). The decompressed raw files were analyzed by Fiji ImageJ software (https://fiji.sc/). Bacterial swimming trajectories were calculated using the “Trackmate” plugin, which uses particle segmentation, and tracking algorithms (http://imagej.net/TrackMate) (34–36). Cell tracks from each video recording frame were segmented and detected using the Gaussian function (34). Tracking of segmented cell outlines was performed by linking each outline from frame to frame based on the linear assignment problem method (35). Velocities of individual swimming cells were obtained from the trajectories. Very slow or immobile cells or tracks (swimming velocity <1 µm/s) or those less than three frames were excluded from the final data analysis. The represented data are the average measurements from two or more independent experiments.

To determine predator maximal growth and prey decay rates (Rmax) and the time needed to reach them (inflection point [S]), dual prey and Tdt-labeled planktonic predator cultures were monitored at OD600 and with fluorescence, respectively, as above. Values were normalized and plotted using the CurVerR package (37). A one-way analysis of variance (ANOVA)-based Tukey’s honestly significant difference (HSD) test was performed on the Rmax and S averages for the different strains, in R.

### Bacterial two-hybrid interactions

Bacterial two-hybrid analysis of interacting protein partners was carried out using the Euromedex system (a kind gift of Anton Peleg, Monash University) (38). Coding sequences of selected proteins of interest were fused to either of the two complementary fragments (T25 [pKT25] or T18 [pUT18C]) of adenylate cyclase using PCR-based red fluorescence cloning (Table S1) and subsequently co-transformed into *E. coli cyaA*^−^ strain BTH101. Positive interactions were observed via functional complementation of adenylate cyclase fragments and expression of the *lac* reporter at 28°C.

Details of bacterial strains, media, and growth conditions; environmental effects on H-I biofilm development; characterization of EPSs; electron microscopy and confocal microscopy; PCR, gene sequencing, and real-time quantitative PCR (RT-qPCR); DNA manipulations, CdG concentration, and statistical analysis can be found in the supplemental text file.

## RESULTS

### Host-independent strains differ in biofilm formation ability

**Fig 1:**
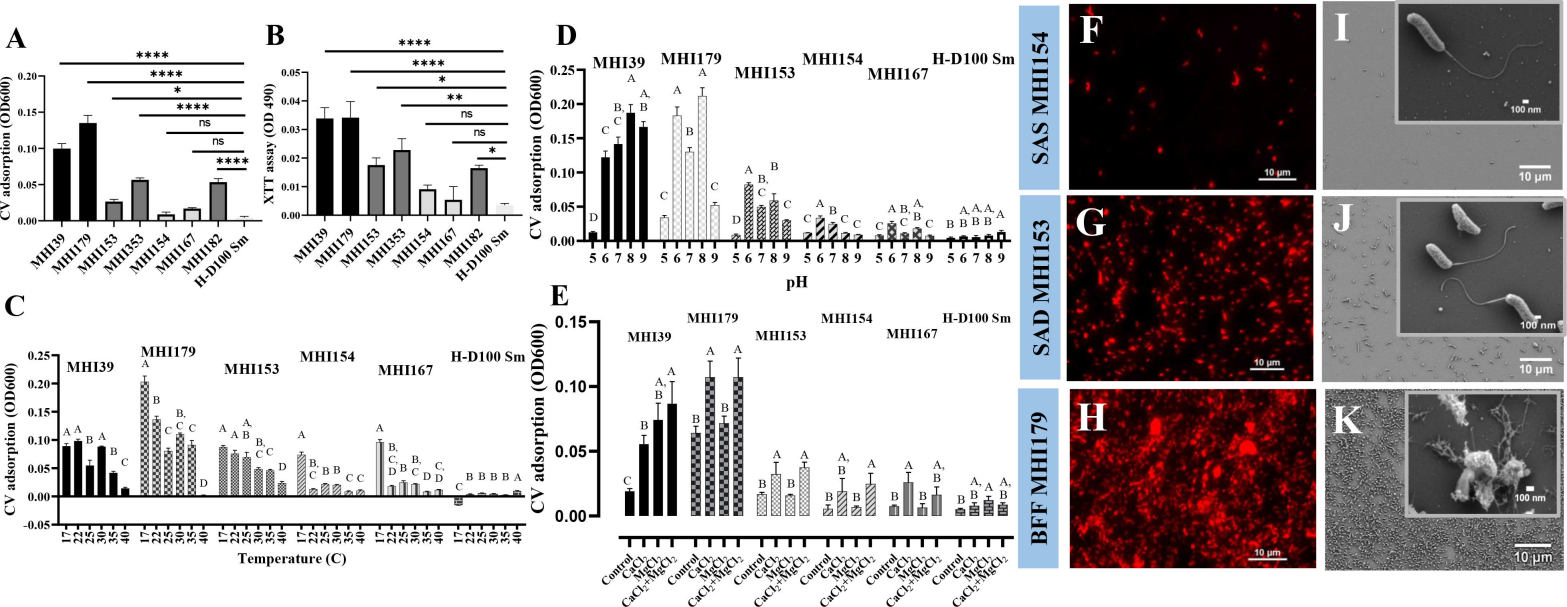
Biofilm formation by H-I strains BFF MHI39 and MHI179; SAD MHI153, MHI353, and MHI182; and SAS MHI154 and MHI167 and by the parental H-D100Sm strain, under various conditions. Biofilms were measured by crystal violet (CV) adsorption (A) and using the XTT cell-respiration assay (B). CV measurements of biofilms at different temperatures (C), pH (D), and concentrations of divalent (Ca^2+^, Mg^2+^) cations (E). Bars with different letters above show categories significantly different by Tukey’s test at *P* < 0.05. Error bars represent standard error of the mean. Microscopic examination of biofilms formed by H-I strains: strains SAS MHI154-tdT (F), SAD MHI153-tdT (G); and BFF MHI179-tdT (H) biofilms viewed by red fluorescence (RF)P epifluorescence. Scanning electron micrographs of biofilms formed by strains SAS MHI154 (I), SAD MHI153 (J), and BFF MHI179 (K). Insets are higher magnifications of a smaller field.

Three hundred fifty H-I colonies were isolated, at a frequency of ~10^−6^. About 25% (86) were Type I, requiring prey extract for growth, and ~75% (264) were Type II, growing on rich medium (19, 28). PCR amplification of *bd0108*, an invasion pole pilus-associated protein (19, 24, 27), confirmed that all were derived from the parental H-D100Sm strain (Fig. S2). Based on crystal violet staining, isolates were categorized as biofilm formers (BFF), surface adherers (SAD), or surface associated (SAS; Fig. S3A). Type-II H-I strains were 17% BFF, 58% SAD, and 25% SAS. Type-I isolates were SAS and did not differ from the parental strain. Following additional staining experiments, two Type-II strains from each class were selected for further analysis (Fig. 1A; Table S2). An XTT cell viability assay confirmed cell activity, concomitant to CV staining (Fig. 1B). Some slight differences were seen between growth curves obtained from the H-I strains in axenic suspension, but they were not related to surface behavior (Fig. S4).

### Temperature, pH, and divalent cations influence surface attachment

In comparison to 30°C, surface attachment was enhanced at low temperature (17°C) by 1%, 84%, 15%, 262%, and 340%, in strains MHI39, MHI179, MHI153, MHI154, and MHI167, respectively, and decreased by 453% in the parental H-D100 strain (Fig. 1C; Fig. S5). At pH 6, compared to pH 8, surface attachment by MHI39, MHI179, and H-D100 decreased by 35%, 13%, and 15%, respectively, but by MHI153, MHI154, and MHI167, it increased by 39%, 194%, and 42%, respectively (Fig. 1D). It was largely reduced at pH 5. Addition of Ca^2+^ to most strains or of Mg^2+^ significantly enhanced surface attachment, with no significant additional enhancement when both were present. No effect was detected in H-D100 (Fig. 1E).

### Surface attachment and biofilm development of SAS, SAD, and BFF strains

**Fig 2:**
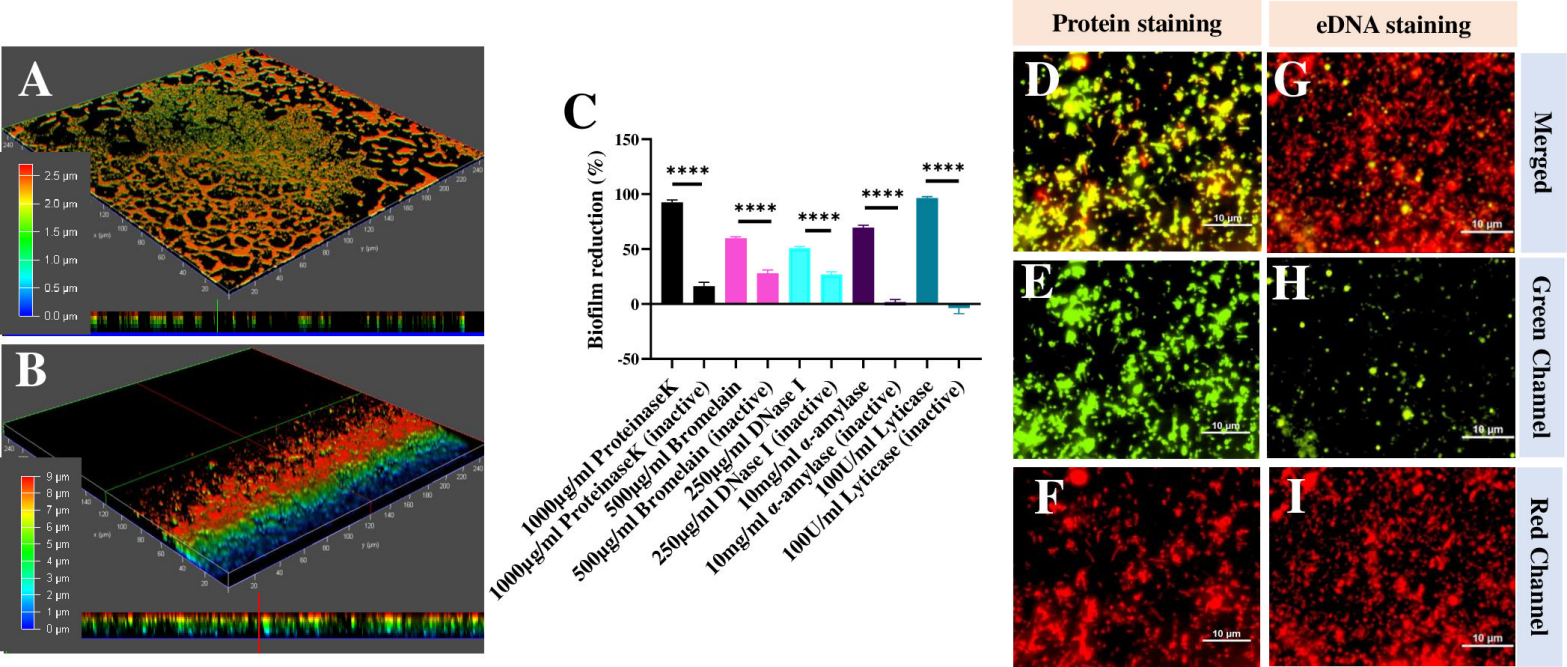
Confocal microscopy-based acquisition of BFF strain MHI179-*tdT* biofilms grown in static culture for 2 days (**A**) and under continuous culture for 7 days (**B**). Biofilm thickness is color-coded based on scale. The bottom part of panels **A** and **B** is an *x-y* axis view, rendering biofilm thickness. Effect of lytic enzymes on biofilm EPS of BFF strain MHI179, and differential staining of matrix components. The percentage of biofilm removal by proteinase K, bromelain, DNase I, α-amylase, and lyticase in comparison to their heat-inactivated counterparts (**C**). The results are averages with stardard errors of three or more experiments. The percentage of biofilm reduction by different enzymes was statistically analyzed by unpaired *t* test comparing the mean of each treatment with the mean of their heat-inactivated counterparts. BFF strain MHI179-tdT biofilm EPS proteins and eDNA stained by FITC isomer I (**D, E, and F**) and DiTO-1 (**G, H, and I**), respectively, as merged (**D and G**), stain only (**E and H**), and tdT (**F and I**) signals.

After 48 hours of incubation on a coverslip, only sparse attachment (1.6% ± 0.3% coverage) of AP-like cells of a SAS (MHI154-*tdt*) strain was observed (Fig. 1F and I; Fig. S6A). A SAD (MHI153-*tdt*) strain achieved denser populations (7.7% ± 0.9% coverage) of both AP-like and elongated cells (Fig. 1G and J; Fig. S6A). Numerous (33.9% ± 1.7% coverage) BFF (MHI179-*tdt*) strain cell aggregates were observed, producing biofilm-like structures, embedded in fibrillar and amorphous-looking material (Fig. 1H and K), reaching up to 4 µm in thickness (Fig. 2A). Biofilm thickness increased to 20 µm when incubated under constant nutrient flow for 7 days (Fig. 2B). No biofilm was formed by the SAS strain within 14 days of incubation while CV staining showed increased interaction of the SAD strain with the surface (Fig. S6B).

BFF MHI179-*tdt* biofilms were treated with proteinase K, bromelain, DNase I, α-amylase, and lyticase, resulting in 92%, 60%, 51%, 70%, and 96% reduction in secreted material, respectively, in comparison to an untreated control (Fig. 2C). Thus, proteins, DNA, and polysaccharides composed the deposited material (Fig. 2; Fig. S7) (23). Extracellular proteins and eDNA co-localized with the Tdt-expressing cell aggregates, with eDNA showing a scarcer distribution (Fig. 2D, E, G, and H). Finally, SEM coupled with energy dispersive X-ray showed cells of different lengths and shapes, indicative of growth and replication, and detected 1%–2% Ca^2+^ in the BFF MHI179 biofilm matrix but no Mg^2+^, in line with the large effect of the former cation on attachment (Fig. 1E; Fig. S8A through G). We conclude that BFFs produce genuine biofilms.

### Predation dynamics of H-I and H-D strains differ

**Fig 3:**
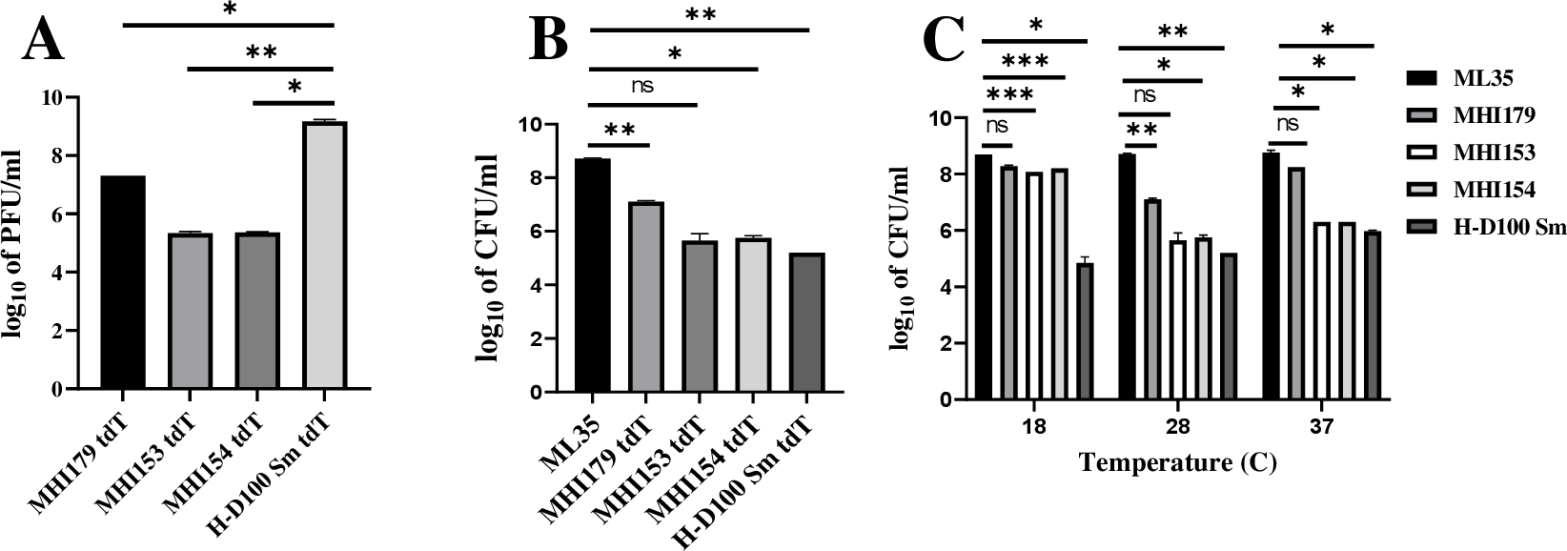
Predatory growth on *E. coli* ML35 of H-I vs H-D100 strain in suspension and temperature effects. (A) Planktonic growth at 28°C of strains BFF MHI179-Tdt, SAD MHI153-Tdt, SAS MHI154-Tdt, and of the parental H-D100Sm-Tdt, measured by plaque forming units (PFU/mL). (B) Prey population reduction, measured by remaining prey counts colony forming units (CFU/mL). The associated growth and prey decay curves showing predation dynamics, maximal growth rates, and inflexion points are presented in Fig. S9 and S10. (C) The effect of temperature on predation by the same strains was measured by remaining prey counts. Error bars indicate starndard error. *E. coli* ML35 without predatory interaction was used as control. The statistical significance of mean values was analyzed by RM (repeated measure) one-way ANOVA with Geisser-Greenhouse correction and *P*-values. ns = *P* > 0.05, * = *P* ≤ 0.05, ** = *P* ≤ 0.01, *** = *P* ≤ 0.001, and **** = *P* ≤ 0.0001.

Predator growth and prey decay parameters were tracked to measure predation dynamics in three H-I strains with different surface attachment capacities (BFF MHI179-*tdt*, SAD MHI153-*tdt*, and SAS MHI154-*tdt*) and in the parental H-D100 strain (Fig. 3; Fig. S9 and S10). H-D100 predator maximal growth rates and associated prey maximal decay rates and the time taken to reach them (inflexion time) were significantly (*P* < 0.05) faster and shorter, respectively, than in the three tested H-I strains (Fig. S9 and S10). Among those, maximal growth rates were similar, but the inflexion times varied, being shortest in SAS MHI154-tdt (*P* < 0.05; Fig. S9). Concomitant prey decay rates and inflexion times were fastest and shortest, respectively, in H-D100 and did not differ significantly between the other strains (Fig. S10).

Also, as determined by plate counts, BFF MHI179-*tdt* achieved higher predator concentrations than SAD MHI153-*tdt* and SAS MHI154-*tdt* (Fig. 3A); yet, remaining prey levels in the dual cultures with BFF MHI179-*tdt* were higher than with SAD MHI153-*tdt* and SAS MHI154-*tdt* (Fig. 3B). Predator and remaining prey populations of all H-I strains were lower and higher than with the parental strain, respectively. When the temperature was decreased to 18°C, H-I strains hardly reduced prey populations. For all strains, prey reduction was maximal at 28°C, but it varied between strains, with prey exposed to BFF MHI179-*tdt* at 28°C and 37°C remaining at the highest levels (Fig. 3C).

**Fig 4:**
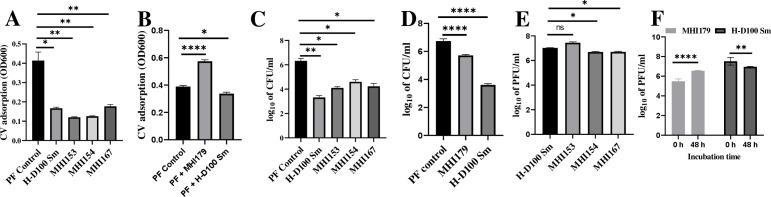
Biofilm formation by *P. fluorescens* and remaining biofilms (**A and B**), predation within the biofilms (**C and D**), and growth of the predators (**E and F**), using strains SAS MHI154, SAS MHI167, and SAD MHI153 (**A, C, and E**), and BFF MHI179 (**B, D, and F**), and by the parental H-D100Sm (A to F). (A and B) Crystal violet biofilm staining. (C and D) Plate counts (CFU/mL) of remaining prey in the biofilms. (E and F) Plaque counts (PFU/mL) of the predators in the biofilms. Error bars indicate standard error. The statistical significance of mean values was analyzed by RM (repeated measure) one-way ANOVA with Geisser-Greenhouse correction and *P*-values. ns = *P* > 0.05, * = *P* ≤ 0.05, ** = *P* ≤ 0.01, *** = *P* ≤ 0.001, and **** = *P* ≤ 0.0001.

Lastly, H-I predation was evaluated in *Pseudomonas fluorescens* prey biofilms. Crystal violet values were reduced by 56%–71% in the SAD and SAS strains but increased by circa 50% in the BFF MHI179 (Fig. 4A and B). As CV staining cannot differentiate between H-I and prey contributions to the biofilm, predator and prey populations were measured directly. Prey cell levels remaining in the biofilm were significantly higher with the H-I strains than with the H-D. Yet, the SAD, SAS, and BFF strains reduced prey levels by at least two and one order of magnitude, respectively (Fig. 4C and D). H-I and H-D100 strains established similar populations within the remaining biofilm (Fig. 4E and F). Thus, SAS and SAD H-I strains were less efficient predators than the H-D100 parent in the planktonic phase, but they were almost as efficient in prey biofilms. The BFF strain preyed less efficiently in both habitats but established high populations and produced EPS in the pre-established prey biofilm.

### Different mutations underlie BFF, SAD, and SAS

We randomly selected, sequenced, and analyzed 44 SAS, SAD, and BFF H-I strains for mutations in *bd0108*, which are known to lead to Type I prey extract-dependent host independency and are found in most H-I strains analyzed so far (27, 39). Although 32% of the strains were mutated in *bd0108*, only 5% of them were SAS, as compared to 82% which were BFF (Table S3). To obtain a larger coverage, four bacterial pools of SAS, BFF, Type-I, and Type-II H-I strains were analyzed by high throughput sequencing. Eleven different *bd0108* variants comprising 21%–32% of the reads were identified in 23% and 32% of BFF and SAS strains, respectively (Table S3). Mutations in RNA degradosome genes *rhlB* and *pcnB* in *bd0108* mutant backgrounds enable robust Type II and BFF phenotypes without prey extract (Fig. S3B). Also, the diguanylate cyclase *dgcC* and the pilus encoding gene *pilB* strongly reduce H-D growth (28, 35, 36,28, 35). Yet, no genetic alterations were detected in these genes (Fig. S11). As a control, the mutation in *rpsL* conferring Sm resistance (28) to the parental strain was observed in all strains, as expected (Table S1; Fig. S11).

As gene-specific sequencing failed to detect genetic variations potentially involved in differential surface attachment and host independency, whole-genome sequencing was performed on two SAS, three SAD, and two BFF strains and on the parental H-D strain. Pairwise comparisons showed each H-I strain to differ from the parent by 1–4 mutations (Table 1). One event was a precise inversion of 48,880 bases adjacent to a two-base GG to AT substitution, another was a 118-base deletion, and the other 12 were single-base substitutions, insertions, or deletions. Thirteen of the 14 mutations were in protein-coding regions, and one was in a promoter binding site (Fig. S12). Each of the sequenced strains had mutations independent from each other, apart from the two BFF strains that shared one silent and one non-synonymous substitution and differed by additional frameshifts in two other genes.

*bd0108* was truncated by different frameshifts and the aforementioned inversion, in the two BFF and two of the three SAD strains. Additionally, 1–3 genes were mutated in these strains (see below). Mutations in stator components *bd3254 motA* (*motA3*) and in *bd1076 fliL* were identified in SAD MHI153, and in SAS MHI154 and MHI167, respectively. In SAS MHI167, a single base substitution in the FliA promoter binding site (40) (Fig. S12) prevented *fliL* expression (Fig. S13A). *bd1076* mutations were confirmed by single-gene sequencing (Table S1). Ten additional SAS strains were screened for mutations in *bd1076* and its promoter region, finding a deletion (ΔT272) in strain MHI155, which truncated the Bd1076 protein. The fully-sequenced SAD MHI153, SAS MHI154, and SAS MHI167 strains had no other mutations. Thus, Type II H-I phenotypes emerge from different mutational paths, including single mutations in flagellar stator genes.

**TABLE 1:** Sequenced H-I strains variations^*a*^

Strain	Biofilm^*b*^	Mutated genes^*c*^								
		bd0019	bd0054	bd0108	bd0149	bd0974	bd1076	bd2152	bd2221	bd3254
MHI39	BFF			c.107_108insA fs 35 + 2	c.767G > T G256V			c.139T > C Silent		
MHI179	BFF	c.91del fs 30 + 13		c.221del fs 73 + 35	c.767G > T G256V			c.139T > C Silent		
MHI182	SAD		g.48168_97049inv, g.97050_97051GG > AT^*d*^							
			fs 108 + 58	fs 42 + 366						
MHI353	SAD			c.98_99insC fs 33 + 4		c.577A > G K193E			c.388_389insA; fs 129 + 44	
MHI153	SAD									c.616_617insC; fs 205 + 3
MHI154	SAS						c.446_507 + 56 del fs 148 + 11			
MHI167	SAS						c.-28G > A Transcript inactivation^*e*^			

^*a*^ DNA and protein variations shown as follows. DNA changes are prefixed by coding sequence “c” or genome “g” prefixes, followed by the gene or genome position/s, respectively. A “−” or “+” preceding a coding position denotes a change in 5′ or 3′ UTR, respectively. Substitutions are shown as “position reference >substitute.” Insertions are denoted as “ins” followed by the insert, deletions are denoted as “del,” and inversions are denoted as “inv.” Protein changes (gray background) are “Silent” for synonymous substitutions, substitutions are shown as “reference aa-position substitute,” frameshift truncations are denoted by “fs,” length of the remaining N-terminal aa + the aa length of the new resulting C-terminal end.

^*b*^ Biofilm forms are BFF, SAD, or SAS.

^*c*^ Genes function: Bd0019: ppiD. Parvulin-like peptidylprolyl isomerase; Bd0054: metallophosphatase; Bd0108: lifestyle switch protein; Bd0149: predicted hypothetical protein; Bd0974: 3-methyl-2-oxobutanoate dehydrogenase; Bd1076: fliL, flagellar basal body-associated protein; Bd2152: glycerol-3-phosphate transporter; Bd2221: Na/Pi-cotransporter family protein; Bd3254: MotA3, flagellar motor stator protein.

^*d*^ The inversion and adjacent two base substitution are between Bd0054 c.324 and Bd0108 c.127. The coordinates correspond to the genome sequence of *B. bacteriovorus* strain H-D100 GenBank accession BX842601.

^*e*^ The single-base substitution is in a conserved base of the −10 box of the predicted FliA-binding site in the promoter of gene Bd1076 (Fig. S12). We experimentally validated the inactivation of transcription in this strain (Fig. S13A).

### Bd0108 complementation in a BFF strain

The biofilm-forming strain MHI39 was complemented with the parental *bd0108* allele to examine the coupling of the H-I phenotype to biofilm formation (Fig. S14). Complemented strains exhibited only prey-dependent growth and failed to grow axenically. A biofilm-forming assay found the complemented strains to have negligible staining, similar to that of the H-D100Sm strain.

### Phylogenomics of stator genes in BALOs

Examination of 16 fully sequenced diverse BALO genomes showed that each includes three *fliL* genes and 1–4 *motAB* gene pairs in separate clusters (Table S4). Specifically, *B. bacteriovorus* H-D100 has three *fliL* and three pairs of *motAB* clusters (Table S5) (41). Altogether we identified 52 flagellar genes in three large clusters with 12 flagellar genes each and 10 small clusters of 1–2 genes totaling 16 genes (six coding for the FliC flagellin; Fig. S15A; Table S5). Two of the *fliL* genes were not adjacent to other flagellar genes, while the third was in a large flagellar class II operon (42). *Halobacteriovorax marinus* SJ flagellar gene organization was very similar to that of *Bdellovibrio*, with three large clusters of 12 flagellar genes each, including a *fliL* gene in a corresponding flagellar class II operon, two stand-alone *fliL* genes, and one *motAB* pair (Fig. S15B; Table S5).

### FliL-Bd1076 affects biofilm formation and prey dependency

**Fig 5:**
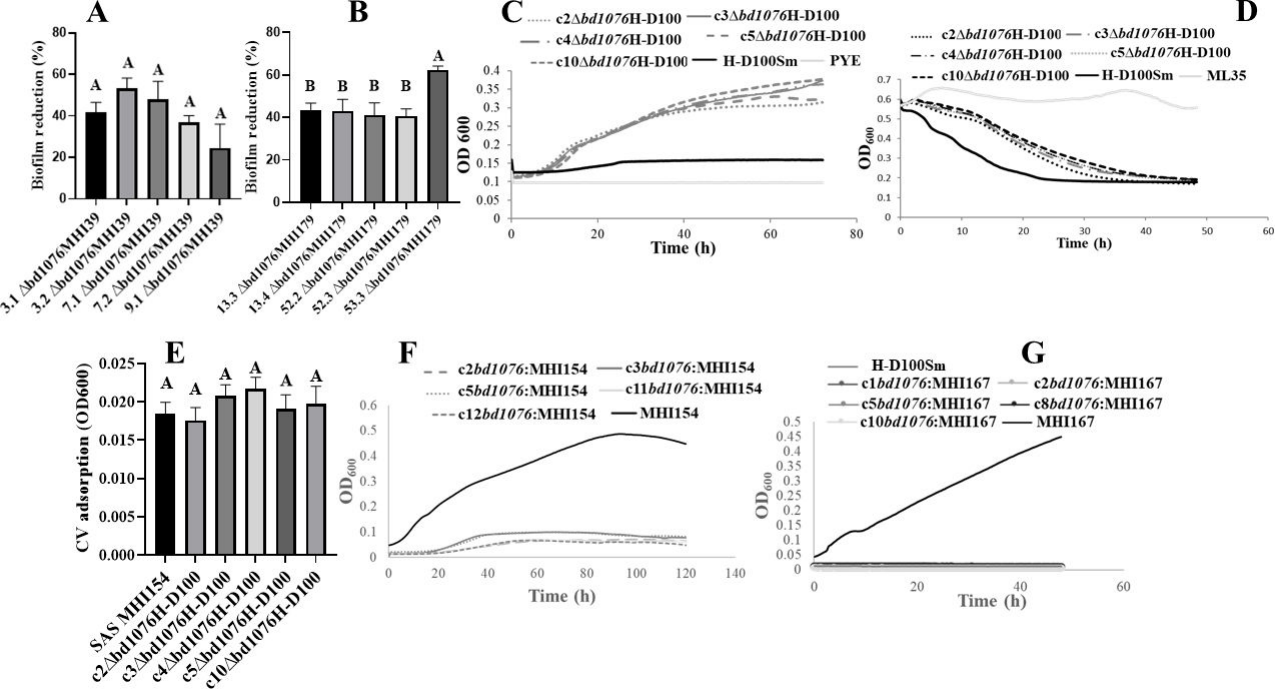
Phenotypes of Δ*bd1076-*deleted biofilm-forming (BFF) MHI39 and MHI179 strains, and native *bd1076*-complemented strains, and of surface-associated (SAS) MHI154, in comparison to the parental H-D100Sm strain. Percentage of biofilm reduction in clonal lineages (identified by numbers) of (A) Δ*bd1076*MHI39 and (B) Δ*bd1076*MHI179; compared to their respective parental strain (100%). (C) Growth of Δ*bd1076*H-D100Sm strains in PYE medium. (D) Predation on *E. coli* ML35 by Δ*bd1076*H-D100Sm strains. (E) CV measurement of biofilm formation by Δ*bd1076*H-D100Sm strains. (F) Growth of *bd1076*-complemented *bd1076*; (G) of *bd1076*-complemented *bd1076*:MHI167, in PYE medium. The results are averages and SEs of three or more experiments (except SE values that are omitted from predation and growth curves). Bars with different letters above show categories significantly different by Tukey’s test at *P* < 0.05.

Next, *fliL-bd1076* was deleted from BFF strains MHI179 and MHI39. Substantial biofilm reduction (24%–62%) was observed in Δ*fliL* mutants vs the parental BFF strains (Fig. 5A and B). To confirm the role of Bd1076 in generating the H-I phenotype and its effect on biofilm formation, *bd1076* was deleted in the obligate H-D100Sm parent. Δ*bd1076*H-D100Sm strains grew host-independently in PYE, exhibited a Type II phenotype (Fig. 5C), and preyed upon *E. coli* less effectively than H-D100Sm (Fig. 5D). Moreover, Δ*bd1076*H-D100Sm mutants were SAS, unable to produce biofilm-like structures (Fig. 5E). Finally, the native *bd1076* allele was inserted into the SAS MHI154 chromosome. Complemented strains were unable to grow axenically but were retrieved as prey (*E. coli* ML35)-dependent cultures (Fig. 5F). Sequencing showed that they contained the parental *bd1076* allele (Fig. S16). The complementation of SAS MHI167 with the native upstream promoter of *bd1076* similarly transformed it from an H-I to an obligate H-D strain (Fig. 5G; Fig. S13B). Thus, FliL-Bd1076 is necessary to maintain the H-D phenotype and significantly increases H-I biofilm development.

### H-I strains are differently affected in motility

**Fig 6:**
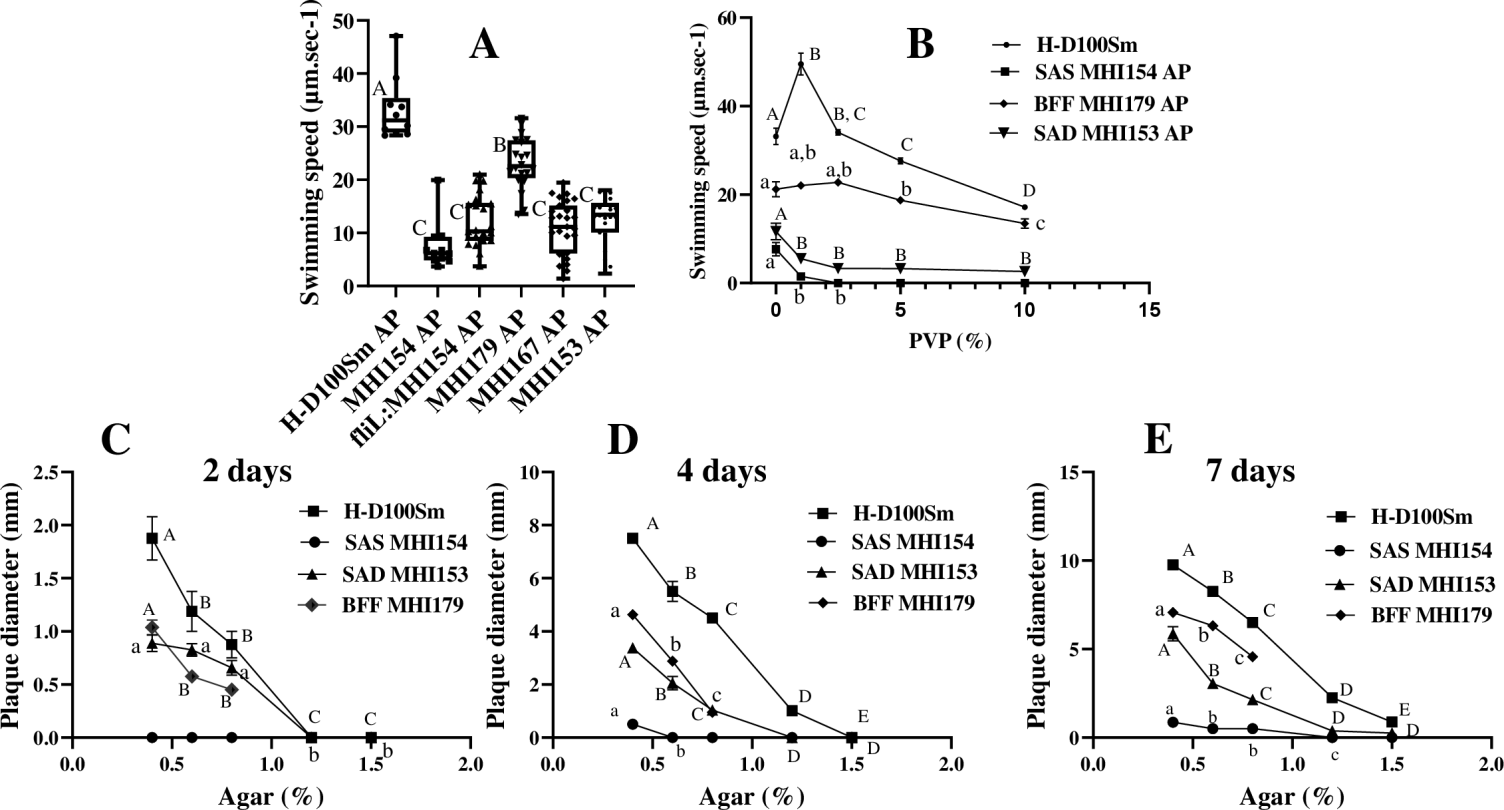
Swimming and swarming motility of H-I and parental H-D100Sm strains. Swimming speed (μm/s) of AP cells in amHEPES (**A**) and in PVP-supplemented amHEPES (**B**). Plaque diameter (mm) of H-D100Sm, SAS MHI154, SAD MHI153, and BFF MHI179 after 2 days (**C**), 4 days (**D**), and 7 days (**E**) of incubation. Values are the mean along with the SEs. In each line and box plot, different letters mark significant differences by Tukey’s test at *P* < 0.05.

H-I AP cells swam significantly slower than H-D100Sm AP cells (Fig. 6A). Velocity was the lowest in SAS MHI154 and MHI167, and SAD MHI153, which were mutated in *bd1076*, in its promoter, or in *motA3*, respectively (Fig. 6A). SAS MHI154 swam very slowly in liquid medium thickened with 1% PVP, and swimming was completely abolished at 2% PVP. SAD MHI153 swimming was reduced to a lesser extent (Fig. 6B). In contrast, parental H-D AP cells were motile even in 10% PVP (33). Although BFF MHI179 was mutated in genes unrelated to flagellar motility, it also swam slower than H-D100Sm, but overall, its swimming speed was little affected by increased viscosity (Fig. 6B). SAS MHI154 hardly formed plaques under increasing agar concentrations, while SAD MHI153 and BFF MHI179 were intermediate as compared to H-D100Sm (Fig. 6C through E). Thus, mutants in stator components FliL-Bd1076 and MotA3 respond differentially to motility challenges.

### Stator-mutant expression changes in genes related to predation and CdG

Based on motility assessment and earlier reports (43, 44), we posited that the *fliL-bd1076* mutation could influence the expression of other genes. Accordingly, 10 flagellum structural and machinery proteins were selected, guided by predicted protein-protein interactions with Bd1076 (Fig. S17). Expression of all 10 genes was substantially upregulated in both prey-dependent and axenic cultures of the Δ*bd1076* mutants SAS MHI154 and MHI167 as compared to the parental H-D strain (Fig. S18A and B). In both strains, large changes were measured in *motA2-bd3021* and *motB2-bd3020* expression (Fig. S18B), while the Δ*bd1076* gene was downregulated in the latter, respectively, as expected (Figure S18B and S13A). Other genes affecting predation like chemotaxis, CdG synthesis, prey-invasion, gliding-related motility, and flagellum modulation (45–47) also exhibited deregulated expression in both SAS MHI154 and MHI167 grown axenically, as well as in MHI154 grown with prey (Table S6; Fig. S18). Lastly, in SAS MHI154, CdG concentrations in AP planktonic populations filtered from PYE-sustained axenic cultures were significantly higher than those of AP populations grown with prey (i.e., in the buffer; Fig. S19).

### FliL Bd1076 specifically interacts with MotB3

A two-hybrid assay was performed to identify flagellar stator proteins interacting with FliL-Bd1076. Bd1076 interacted with itself and with MotB3-Bd3253 (Fig. S20). However, no FliL-Bd1076 interaction was detected with MotA3-Bd3254, MotA2-Bd3021 or MotB2-Bd3020, or between MotA3 and MotB3, and between MotA2 and MotB2, suggesting specific interactions due to sequence differences (Fig. S21).

## DISCUSSION

H-D BALOs are obligate predators, unable to replicate as free-living cells. Therefore, they are incapable of forming biofilms by their own. We find here that Type II H-I and facultative predator strains with *bd0108* mutant backgrounds can develop robust biofilms. In contrast, single mutations in stators genes promoted H-I but impaired biofilm formation. Thus, H-I growth is necessary but not sufficient for biofilm formation.

Stators channel ion motive force to power flagellar rotation (48). MotA and MotB units form the flagellum stator ring complex, spanning the periplasmic space, from the inner membrane to the peptidoglycan layer (49, 50). This ring is integrated with a FliL ring that stabilizes the MotB-peptidoglycan interaction, facilitating ion flow (49, 50). This in turn increases torque, likely by relieving inhibition of proton flow by the MotB plug domain (51). Thus, the absence of *B. bacteriovorus* FliL*-*Bd1076 may directly affect torque generation and motility or change the number of stators, with similar results (52). Furthermore, in a number of bacteria including BALOs (33, 53–55), swimming velocity increases at low viscosities through the interaction of flagellar rotation with the viscosity-inducing polymer (54, 56). FliL has a crucial role in ﬂagellar regulation under increased viscosity in *Bradyrhizobium diazoefficiens* (57), and in flagellar filament release on swarm agar in *Salmonella enterica* (58). We found strong motility disabilities, even at low viscosities, in strains mutated in *fliL*, demonstrating a prominent role for FliL in maintaining stator stability in *Bdellovibrio*. Noteworthy, the SAD MHI153 *bd3254-motA* mutant motility phenotypes were significantly milder. Although strain BFF MH179 mutations in genes *bd0108*, *bd0019*, *bd0149*, and *bd2152* seem to be unrelated to motility, its swimming in liquid and its motility in semi-solid media were also reduced, showing along with previous studies that host-independency impairs motility (59, 60). Yet, low motility did not prevent predation (41, 61). Moreover, while slower growth and predation dynamics, as well as lower and higher final predator and prey levels, respectively, suggest a tradeoff between the H-D and the H-I forms in planktonic predation, motility deficiencies alone do not explain the differences in predation behavior between the strains. Differences in remaining prey populations after predation by the different strains and under the different growth conditions may be related to uninheritable, reversible plastic resistance to BALOs previously observed in suspensions and biofilms (9, 62). Plastic resistance varies between predator and prey combinations and is possibly influenced by population dynamics and the interaction of the predator with the prey (63–65). A very recent publication showed that several fiber-like proteins participate in prey epitope recognition and invasion (66) and may thus also affect predator dynamics in H-I mutant backgrounds. The potential tradeoff appeared weaker in prey biofilms, as predator and remaining prey population sizes were quite similar, maybe because predator-prey proximity in biofilms may lessen the necessity for high motility (61). Yet, BFF MHI179 reduced the prey population to a lesser extent while contributing to the biofilm matrix, maybe using part of the prey matrix as nutrients (10). In contrast, SAD MHI153, which showed increased CV staining under long incubation, did not contribute to the biofilm. These data collectively lead us to propose that a BFF phenotype may be advantageous to invade natural, complex biofilms, widening the bacterium’s niche under adequate conditions.

*B. bacteriovorus* H-D100 has three FliL and three MotAB pairs (41) encoded in individual operons with each MotAB pair probably forming a specific stator unit type. Different amounts of stator units and types can be present in stator rings (67) and might interact differently with FliL proteins. However, our interaction analyses showed that FliL-Bd1076 specifically interacted with itself and with MotB3-Bd3253 but not with the tested MotA proteins, in accordance with structural models (48–50). This may explain the lack of functional complementation in Δ*fliL-bd1076* and Δ*motA-bd3254* even though the other *motA* and *fliL* alleles were upregulated. This agrees with prior findings, showing a lack of complementation by *B. bacteriovorus motAB* genes in *E. coli* (41). Our phylogenomic analysis revealed that in BALOs, the ratio of FliL to MotA-MotB alleles varies from 3:3 to 3:1. Except for one *fliL*, these alleles stand single or as gene pairs, in contrast to almost all other flagellar genes which are organized in large clusters. Separate regulation of stator genes may enable fast and independent reactivity of stator modules, providing an additional level of ecophysiological flexibility, as BALOs adapt their swimming to prey availability and environmental conditions (33, 47, 68).

Remarkably, a *fliL*-Δ*bd1076* mutation introduced into the BFF MHI179 background significantly reduced biofilm formation, uncovering a hitherto unknown positive effect of FliL on biofilm formation, in line with a role for stator components in signal cascades that ultimately control flagellar gene transcription and bioﬁlm formation (69–71). In *Pseudomonas aeruginosa* and *Vibrio cholerae*, interacting Mot and Dgc proteins maintain high CdG levels during the initial stages of surface colonization, in turn activating biofilm production (72, 73), while in *Caulobacter crescentus*, FliL interacts with the YcgR flagellar brake homolog receptor DgrA to block motor function (74). In our stator gene mutant strains, genes *dgcA* and *dgcB* (which are necessary for H-D growth, gliding and swimming) were upregulated. In contrast, *dgcC*, a gene required for H-I growth, and *merRNA* (a predicted CdG riboswitch expressed at very high levels in H-D AP but at low levels in GP) were downregulated (40, 45, 75, 76), as were the YcgR flagellar brake CdG-effectors homologs *bd0760* and *bd1007* that control swimming during AP (47). Together with the very large difference in CdG concentrations in SAS MHI154 AP cells from axenic vs prey-dependent cultures, these data clearly show that stator disruption modifies CdG fluxes.

**Fig 7:**
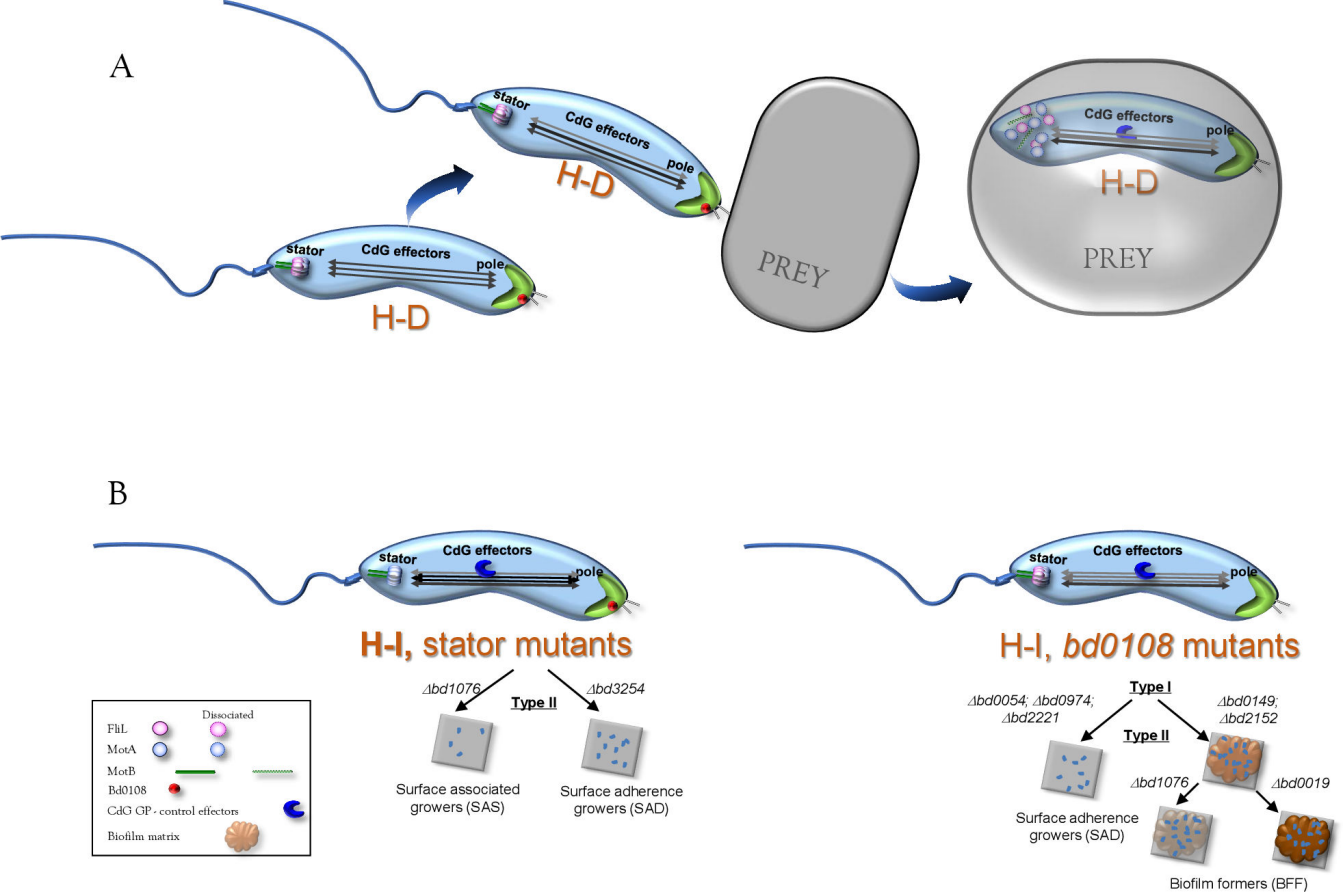
Conceptual schematic model of possible signaling cascades leading to different H-I phenotypes emerging from mutations in stator genes or in *bd0108*. (A) Parental strain: upon prey binding, CdG fluxes change between the invasion and flagellar poles, bringing upon changes in gene expression (29). Prey invasion leads to the dismantling of the flagellar machinery (77), along with further changes in CdG fluxes, and induction of the GP. (B) In stator gene mutants, the stator is destabilized, altering CdG fluxes in the AP and the action of GP control proteins. The mutants are Type II, and poorly associate (surface-associated, SAS) or adhere (surface-adhering, SAD) to surfaces but do not form biofilms (left side). *bd0108* mutations form Type I H-I strains unable to form biofilms. Additional mutations result in Type II strains, which adhere (SAD) or form biofilms on surfaces (biofilm formers [BFF]). Stator genes are necessary for strong biofilm growth (right side, with biofilm intensity indicated by darker colors).

We remark that during prey invasion, the flagellum machinery of *B. bacteriovorus* is internalized and degraded, breaking down the motor before GP (77). We propose that motor breakdown modifies CdG fluxes, in turn—at least partly—controlling the onset of GP. Accordingly, stator-destabilizing mutations (as in *fliL* or *motA*) may similarly bring about a shift in GP control, promoting H-I growth (Fig. 7). In other bacteria, surface sensing is transduced to cellular responses by cAMP and CdG effectors, with cAMP-CRP domains affecting bioﬁlm formation through carbon metabolism (78, 79). Potential candidates for similar roles in *B. bacteriovorus* include the putative CdG effectors CRP and cAMP-CRP proteins Bd0081, Bd1971, and the cAMP-EAL Bd2590 (80, 81) which were deregulated in stator gene mutants strains.

Both BFF strains exhibited robust Type II growth and produced EPS, composed of extracellular proteins, eDNA, and polysaccharides. EPS secretion was promoted by low temperature—a feature shown in *V. cholerae* to be CdG-dependent (82), and by calcium; perhaps increasing matrix stability and cohesiveness and preventing cell dispersion (83, 84). Both strains were mutated in *bd0108*, a gene located just downstream of a large Type IVb pilus operon (85, 86) that codes for a disordered protein linking Type IV pili activity to the invasive pole complex (19, 27, 46). This complex includes a CdG effector, potentially linking prey sensing to flagellar motility through CdG signaling (46).

*bd0108* mutations result in non-biofilm forming Type I H-I strains that grow poorly in prey extract-amended medium, and additional mutations in RNA degradosome genes *pcnB* or *rhlB* are required to graduate to Type II (24, 28, 39). However, these latter genes were intact in BFF MHI39 and MHI179, which shared the same mutations in *bd0149* (coding for an uncharacterized protein) and in *bd2152* (glycerol-3-phosphate transporter) (40), which thus probably have occurred prior to the *bd0108* mutation. Although the mutation in *bd2152* is synonymous and might be neutral, it alternatively may affect phenotype by altering the DNA to protein cascade (87). BFF MHI179 had an additional mutation in *bd0019*, a putative major periplasmic parvulin-like peptidylprolyl isomerase (PpiD) chaperone (88, 89) a protein shown to increase biofilm formation in *Campylobacter jejuni*. BFF MHI39-derived strains complemented by the parental H-D *bd0108* allele were incapable of H-I growth and were only obtained under H-D conditions, unambiguously demonstrating that while *bd0108* underlied the H-I shift, *bd0149* and *bd2152* were responsible for the Type II phenotype and for biofilm formation. This was further supported by Type II strains SAD MHI182 and MHI353 which were mutated in *bd0108*, and in one (*bd0054*, a membrane metalloprotease), or two (*bd0974*, a methyl-oxobutanoate dehydrogenase; *bd2221*, a Na/Pi-cotransporter) other genes but could not form biofilms. Interestingly, *bd0054* and *bd2221* homologs in *B. bacteriovorus* NC01 were mutated at high frequency in a lab evolution experiment, selecting for improved survival under starvation (90). Together, this demonstrates that robust axenic growth (Type II) and facultative predation are accessible through single mutations in stator genes as well as through mutations of diverse genes in *bd0108* mutant backgrounds, and these mutations form the basis for very different H-I phenotypes. Moreover, the large fraction of non-mutated *bd0108*, degradosome-associated, and stator genes in H-I populations suggests that further routes to host independency are possible.

This high diversity, the high frequency at which H-I strains may be obtained (10^−2^) (91), the isolation of an H-I strain from nature (20), and efficient H-I growth in prey biofilms lead us to propose that host-independency coupled with facultative predation may be beneficial in organic matter-rich habitats (4, 5, 10, 92, 93). Also, molecular data show that BALO proteins essential for predation are common with those of the motility apparatus of facultative predators and evolved from shared ancestors (46, 94, 95). Taken together, we speculate that H-I BALOs are common in nature; they may be locally selected from H-D predators which may—or may not—revert to the previous state, or they may form stable derived groups within the large BALO diversity (5, 96). Lastly, the H-D vs the H-I phenotypes, and differences in the latter, may condition predator-prey dynamics, leading to various outcomes (e.g., stable vs unstable coexistence, extinction) (97). The origin of the H-D to H-I transition and its significance on community dynamics merit further investigation.

## Data Availability

Full genome sequences of the Sm parental strain and its 7 derived MHI isolates were deposited at GenBank (accession codes CP160491, CP160490, CP160485, CP160484, CP160487, CP160488, CP160489, and CP160486). All 8 samples are at NCBI BioBroject PRJNA1127844, with BioSample codes SAMN42024233, SAMN42021370, SAMN42021371, SAMN42021372, SAMN42021373, SAMN42021374, SAMN42021375, and SAMN42021376. Single gene sequences were deposited in NCBI with GenBank accession numbers PQ001029–PQ001055 (*bd0108*), PP960807–PP960811 (*pilB*), PP967218–PP967222 (*rpsL*), PP967223–PP967226 (*dgcC*), and PP960792–PP960804 (*bd1076*). The rest of the data is available upon request

## SUPPLEMENTAL MATERIAL

Supplemental figures mbio.00715-24-s0001.docx Figures S1 to S21.

Supplemental text mbio.00715-24-s0002.docx Additional materials and methods.

Supplemental tables mbio.00715-24-s0003.pdf Tables S1 to S6.

ASM does not own the copyrights to Supplemental Material that may be linked to, or accessed through, an article. The authors have granted ASM a non-exclusive, world-wide license to publish the Supplemental Material files. Please contact the corresponding author directly for reuse.
