# Supplementary material for: Flagellar stator genes control a trophic shift from obligate to facultative predation and biofilm formation in a bacterial predator
Source: mBio. 2024 Jul 22;15(8):e00715-24. doi: 10.1128/mbio.00715-24 (PMC11323537; doi:10.1128/mbio.00715-24)
Supplement: Supplemental figures — Figures S1 to S21. [file mbio.00715-24-s0001.docx]

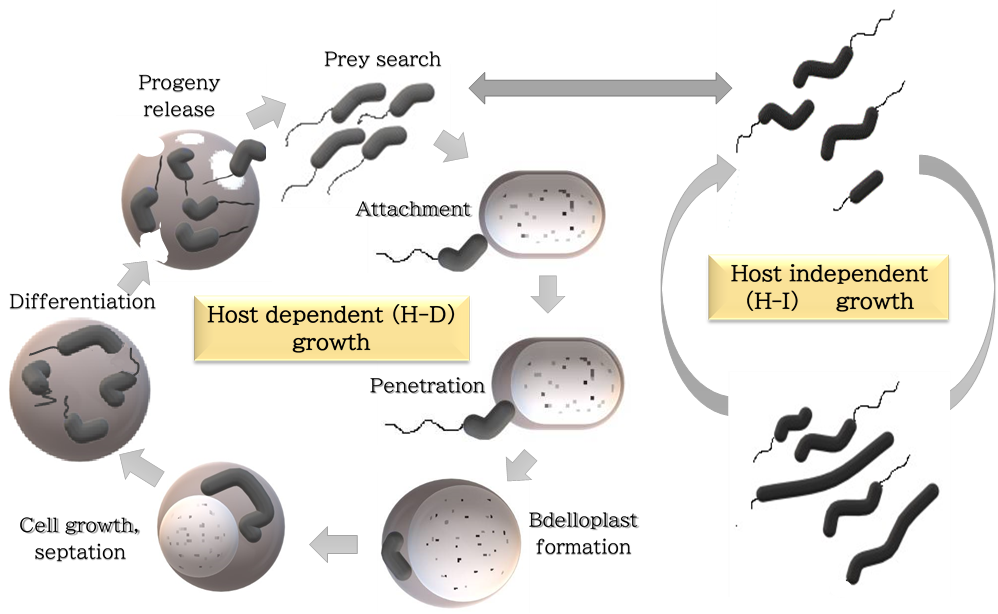


**Figure S1.** Host(prey)-dependent (H-D) and host-independent (H-I) growth cycles of *Bdellovibrio bacteriovorus*.


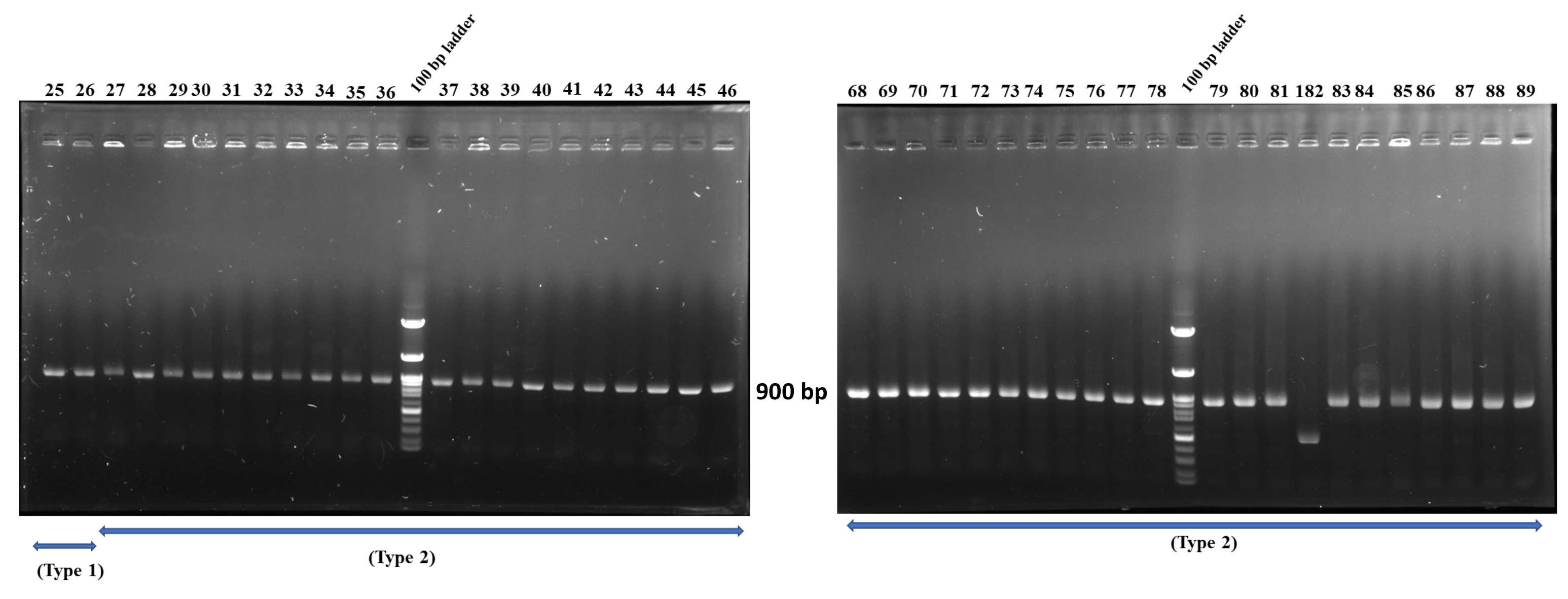


**Figure S2.** Validation of colonies as host-independent (H-I) strains of *B. bacteriovorus,* by PCR amplification of the *hit* locus (*bd0108*)^1^ (3F and 913R primer set, table S1). All the tested strains (numbered above the gels) were positive for the expected product (~900 bp) except MHI82 which includes a deletion in that gene (Table 1). A 100 bp DNA ladder is included in the middle of each of the two gels.

**
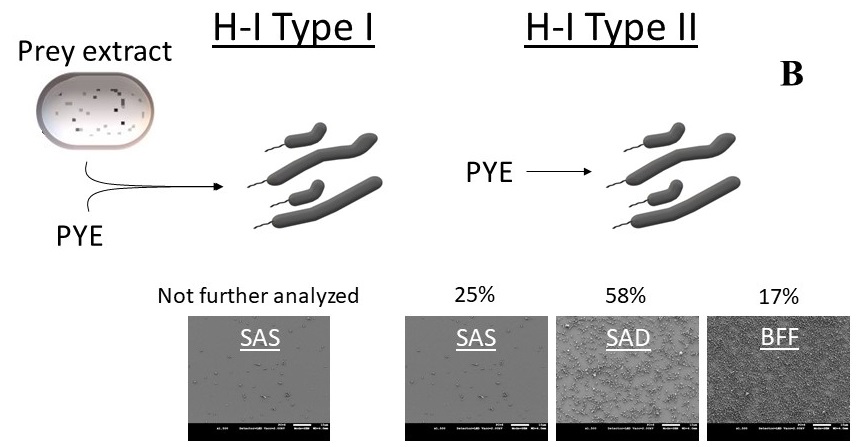

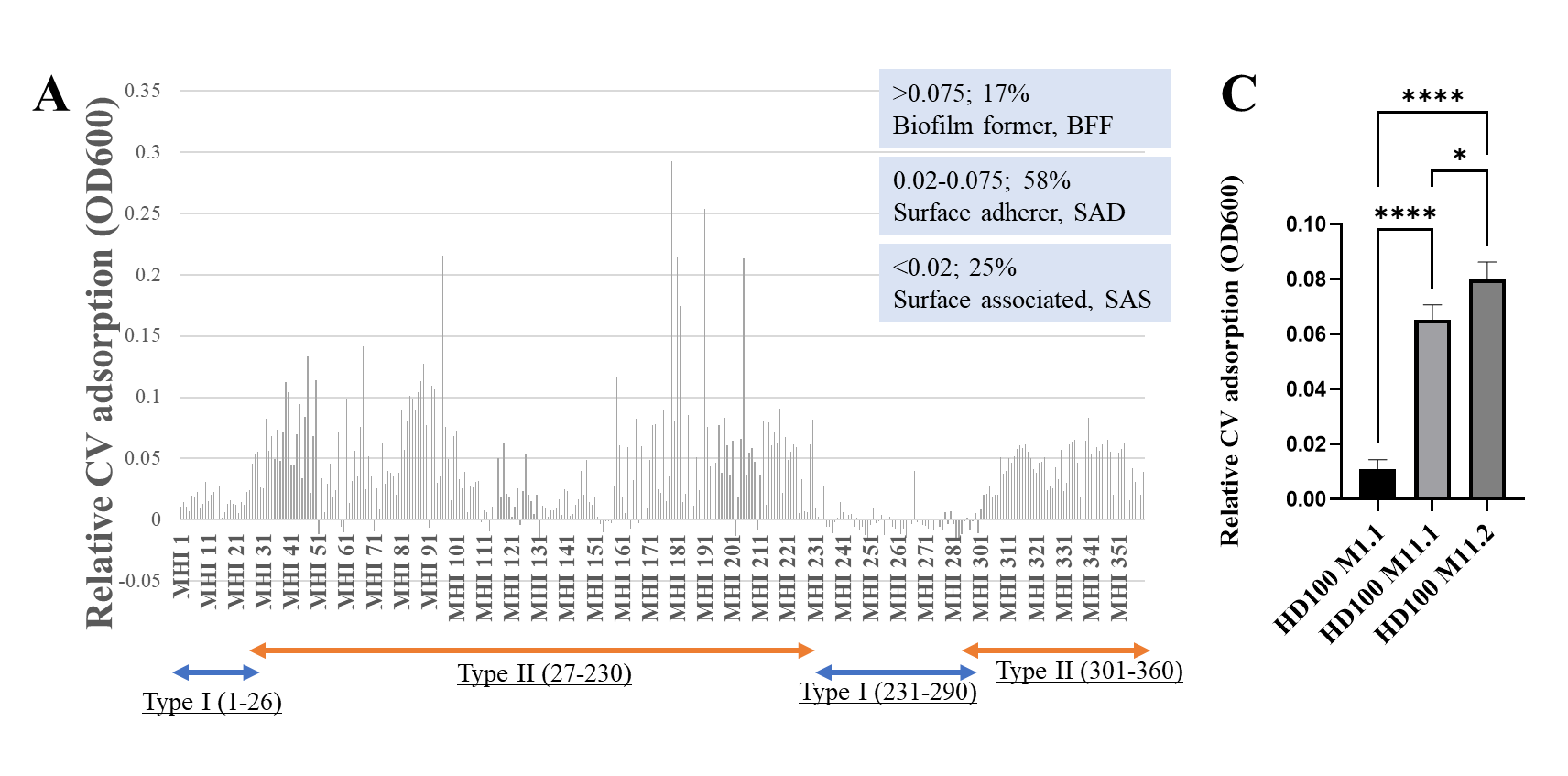
**

**Figure S3.** Crystal violet (CV) staining (OD_600_) of axenic biofilms in polystyrene microtiter plates. A. Formed by newly isolated Type-I and Type II H-I strains (see B). Numbers identify specific strains. B. H-I Type I and H-I Type II growth requirements and the % of strains in panel (A) differing in surface properties as measured by CV staining: Surface associated (SAS, 0.02<CV); Surface adherer (SAD, 0.02<CV<0.075); Biofilm former (BFF, CV>0.075). C. CV staining of M11 H-I mutants bearing a *bdl0108* mutation (Type I), and additional mutations in the RNA degradosome *pcnB* (M11.1) or *rhlB* (M11.2) genes (Type II)^2^. In (C), significant differences in CV values are specified by *=0.05 >p, **** = 0.0001>p , Tukey’s test.


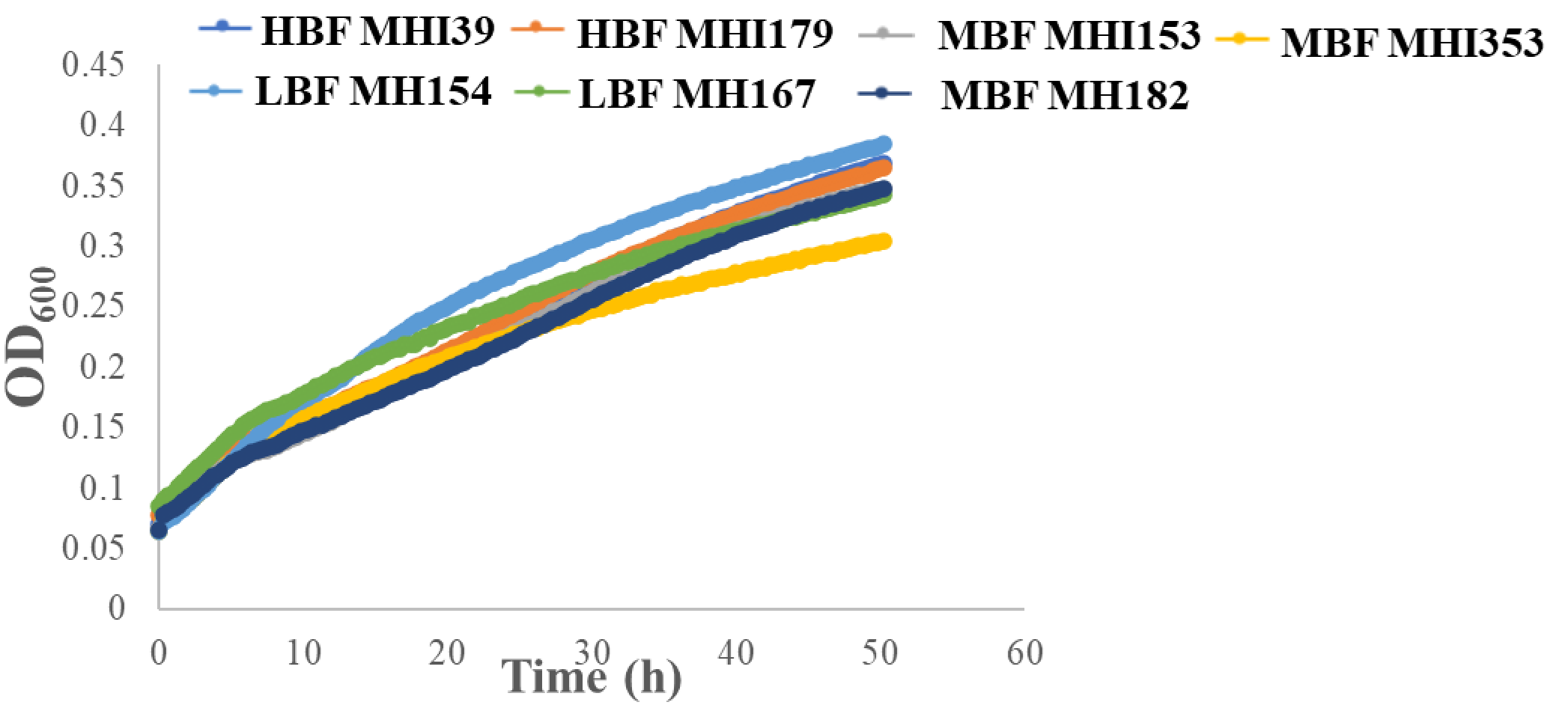


**Figure S4.** Axenic growth of H-I strains BFF MHI39, BFF MHI182, SAD MHI153, SAD MHI35, SAS MHI154 and SAS MHI167 in PYE medium.


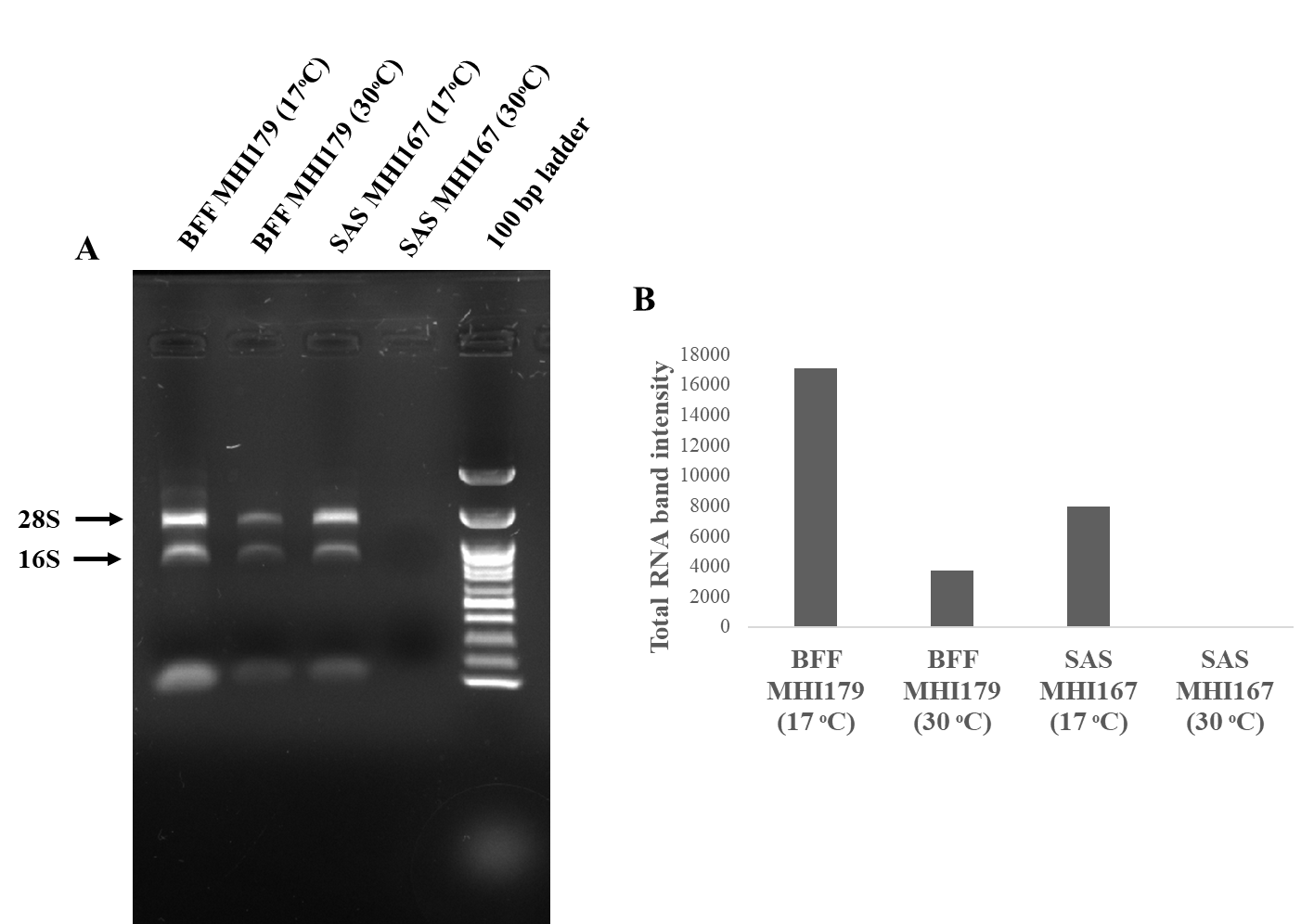


**Figure S5.** Total RNA extracted from H-I strains MHI179 and MHI167 grown as axenic biofilms at different temperatures. A. The RNA extract was electrophorized on a 1% agarose gel for 30 min, and stained with RedSafe™. B. Band intensities. Intensities were acquired by BIORAD Gel Doc XR^+^ with Image Lab^TM^, and measured using ImageJ analysis. The results were based on one experiment.


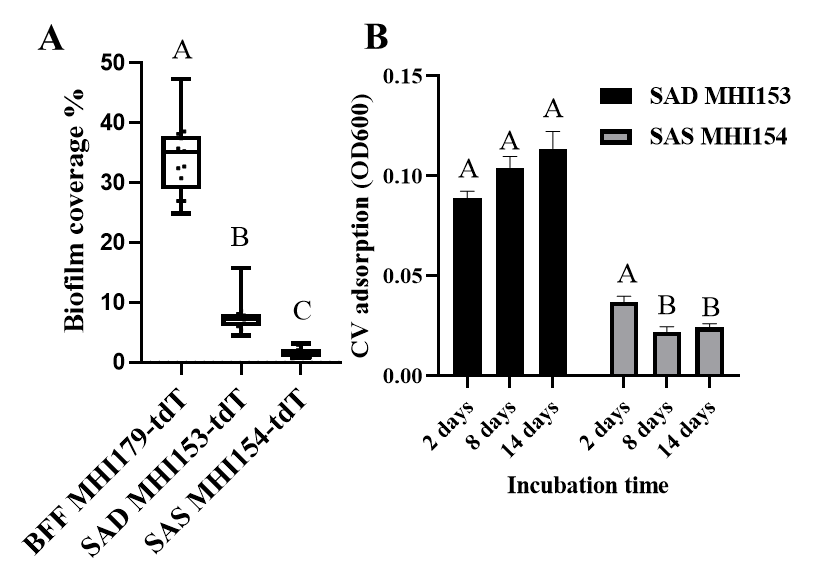


**Figure S6.** A. Surface coverage (in percent) of a polymeric coverslip bottom by surface adhering H-I strains MHI179-tdT, MHI153-tdT and MHI154-tdT, represented by box and whiskers plot. B. Biofilm development by strains MHI153-tdT and MHI154-tdT measured by CV staining after different incubation periods with successive medium change every 2 days. In both (A) and (B) results not labelled by the same letter are significantly different by Tukey’s test at 0.05% significance level. In (B) the statistical analysis was performed independently for the results obtained with MHI153 and MHI154.


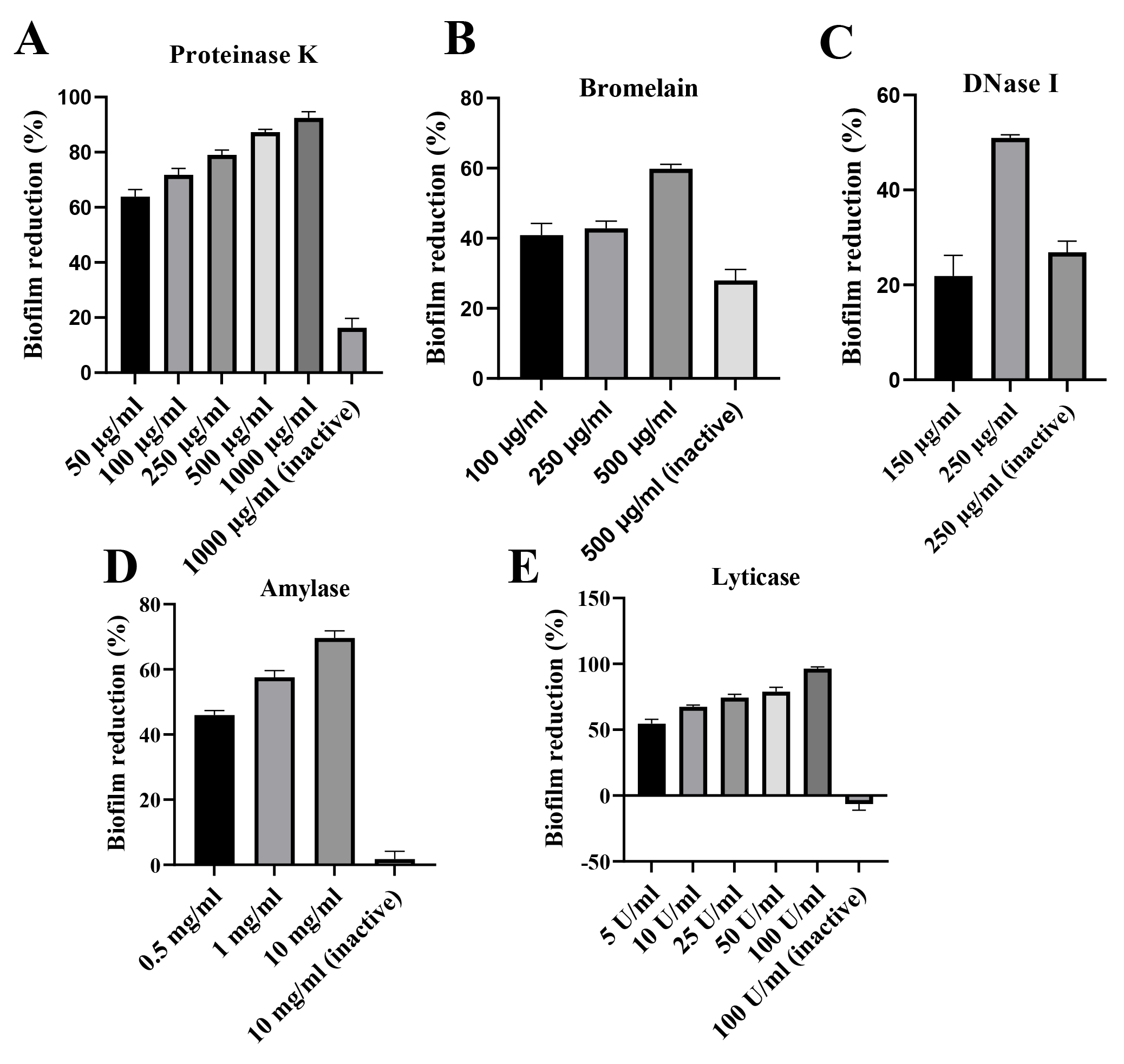


**Figure S7**. Reduction of biofilm formation by H-I strain MHI179 grown axenically in PYE by enzymatic treatments at various concentrations. Proteinase K (A), bromelain (B), DNase I (C), α-amylase (D), and lyticase (E) along with their heat-inactivated controls. The results are averages and standard errors of three or more experiments.


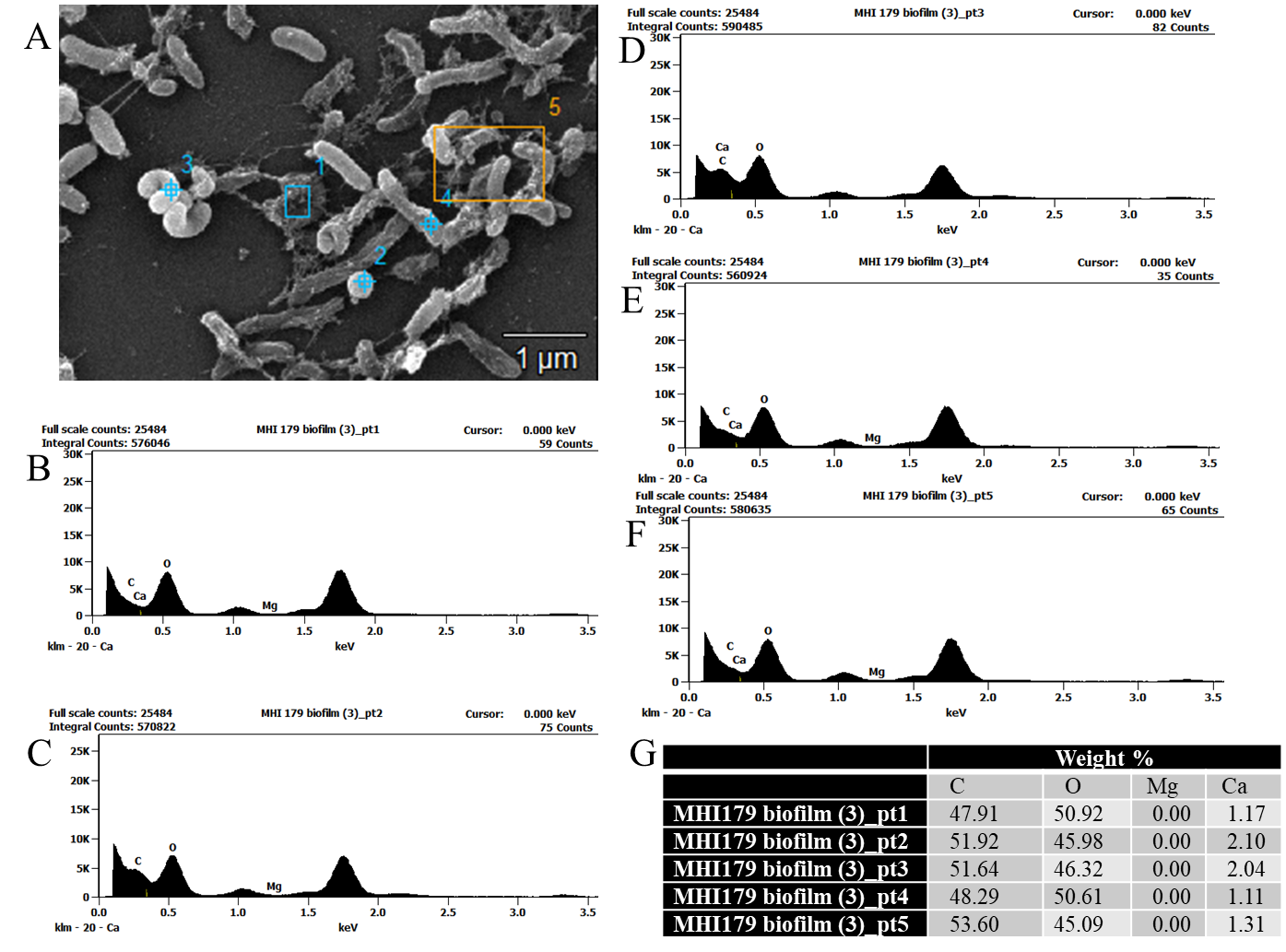


**Figure S8.** SEM-EDX analysis of a strain MHI179 axenic biofilm. A. SEM micrograph of a MHI179 biofilm with selected regions (cyan) where energy-dispersive X-ray spectroscopy (EDS) was performed. B, C, D, E, F, correspond to 1, 2, 3, 4, 5 in (A). G. Percent weight of C, O, Mg and Ca elements detected in the selected regions.

**Figure S9.** Growth rates (Rmax) and inflexion points (S) of strains BFF MHI179, SAD MHI153-*tdt,* SAS MHI154-*tdt*) and of the parental H-D100 growing as dual predator-prey cultures with *E. coli* as a prey (A, B, C, D) Growth was tracked by fluorescence. Significant differences in Rmax and S values between strains are shown as different letters, using one-way Anova-based Tukey’s HSD test (E). Predator only cultures (F, G, H, I), prey alone culture (J), and buffer (K) were used as controls. All cultures together (L).

**Figure S10***. E. coli* prey population decay rates (Rmax) and inflexion points (S) when preyed upon by strains BFF MHI179, SAD MHI153-*tdt,* SAS MHI154-*tdt*) and the parental H-D100 in dual predator-prey cultures (A, B, C, D) Growth was tracked at OD600. Predator only cultures (E, F, G, H), prey alone culture (I), and buffer (J) were used as controls. All cultures together (K). Significant differences in Rmax and S values between strains are shown as different letters, using one-way Anova-based Tukey’s HSD test (L).


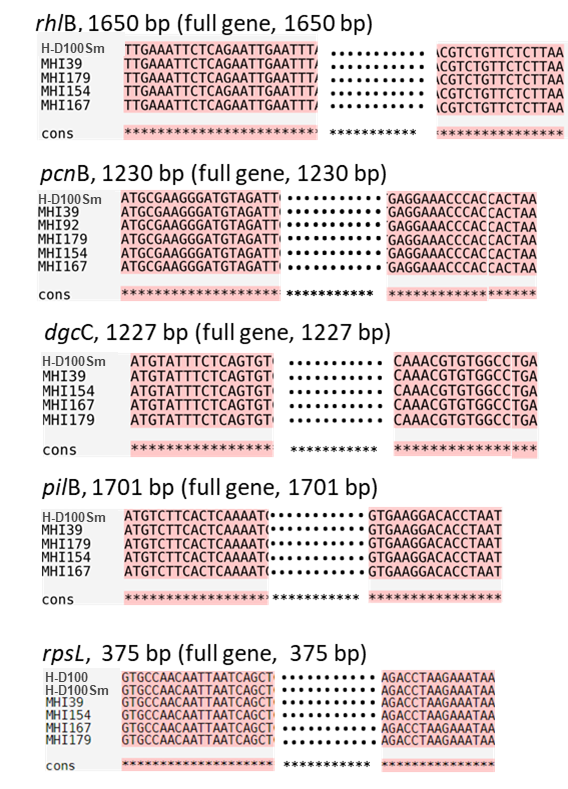


**Figure S11.** Multiple sequence alignmenst of *rhlB, pcnB, dgcC, pilB* and *rpsL* sequences from strains HD100, HD100Sm, BFF MHI39, SAS MHI154, SAS MHI167 and BFF MHI179 using the M-Coffee webtool (https:// tcoffee.crg.eu/apps/tcoffee/do:mcoffee). Only part of the sequences is shown as they were all identical, as indicated by stars. The full sequences are available upon request.

**A**

**B**

**Figure S12**. A. Graphical representation of 30 genomic regions in Bdellovibrionales, between the *Bd1075* and *fliL* Bd1076 genes. Only positions present in H-D100 are shown, omitting insertions in other species. Positions are numbered by their distance away from the *fliL* coding region 5’ start, and also labelled by the H-D100 residue of the position. A G>A mutation in position -28 in SAS MHI167 is marked by a red asterisk; The *fliL* transcription start site (TSS) and FliA sigma-28 bipartite motif at the TSS -35 and -10 are also labelled. B. FliA binding motif from 97 AP promoters based on^3^, labelled as in (A), together with alignment gap positions.


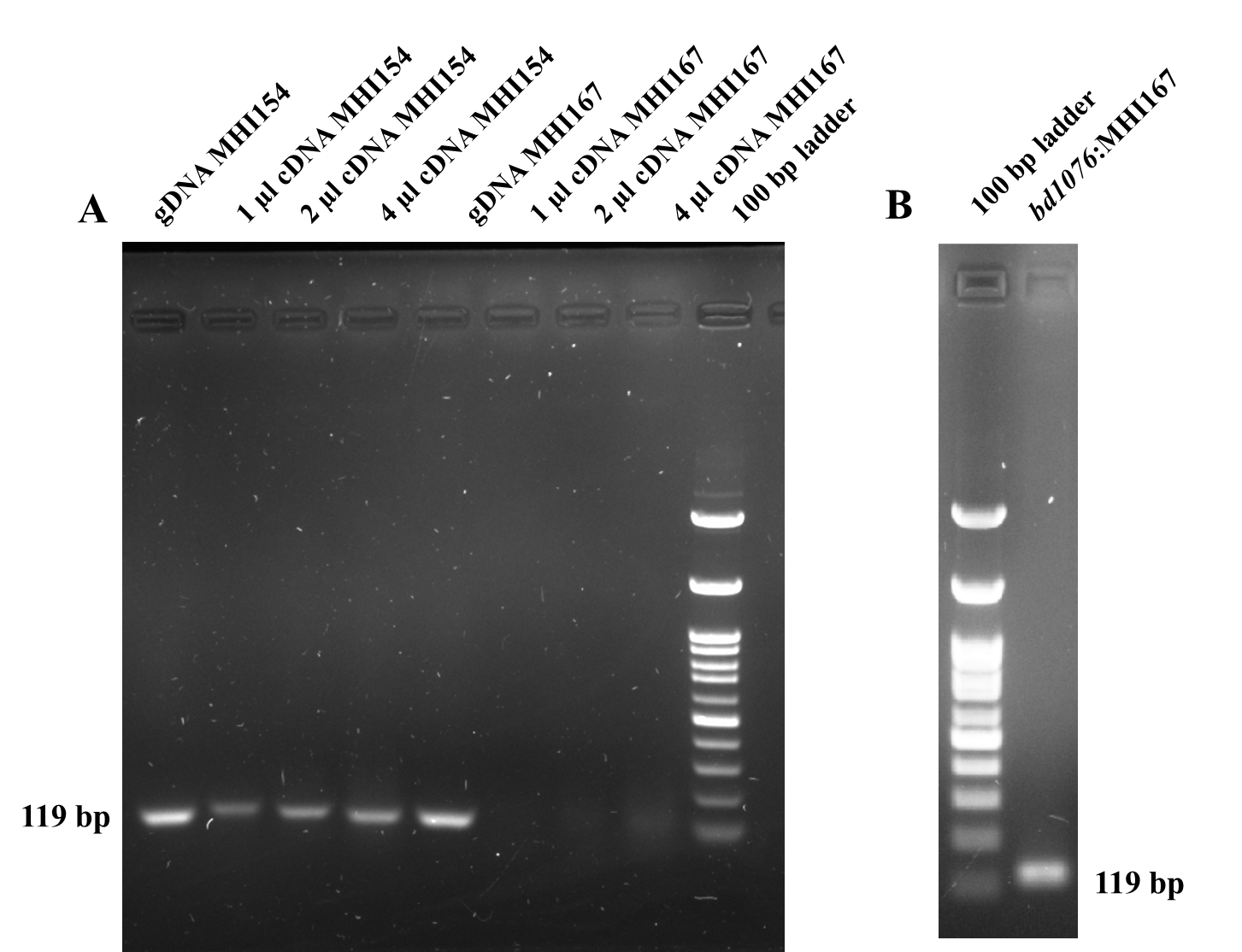


**Figure S13**. A. Gene expression analysis of *fliL* *bd1076* in H-I strains SAS MHI154 and MHI167 using the *fliL*ex primer set (Table S1). B. Gene expression analysis of *bd1076* in *bd1076*:MHI167 strains using the FliLex primer sets with expected product length of 119 bp. A 100 bp DNA ladder is included in the middle of each of the two gels.

**Figure S14**. Crystal violet staining of the MHI39 biofilm-forming strain and two independent complemented strains after introduction of the parental H-D100Sm (HD100) *bd0108* gene. One way anova based Tukey’s HSD test has been used for testing significant difference of CV (OD600) values between MHI39,Complement 1, complement 2 and H-D100. P value < 0.01 = significant (**)


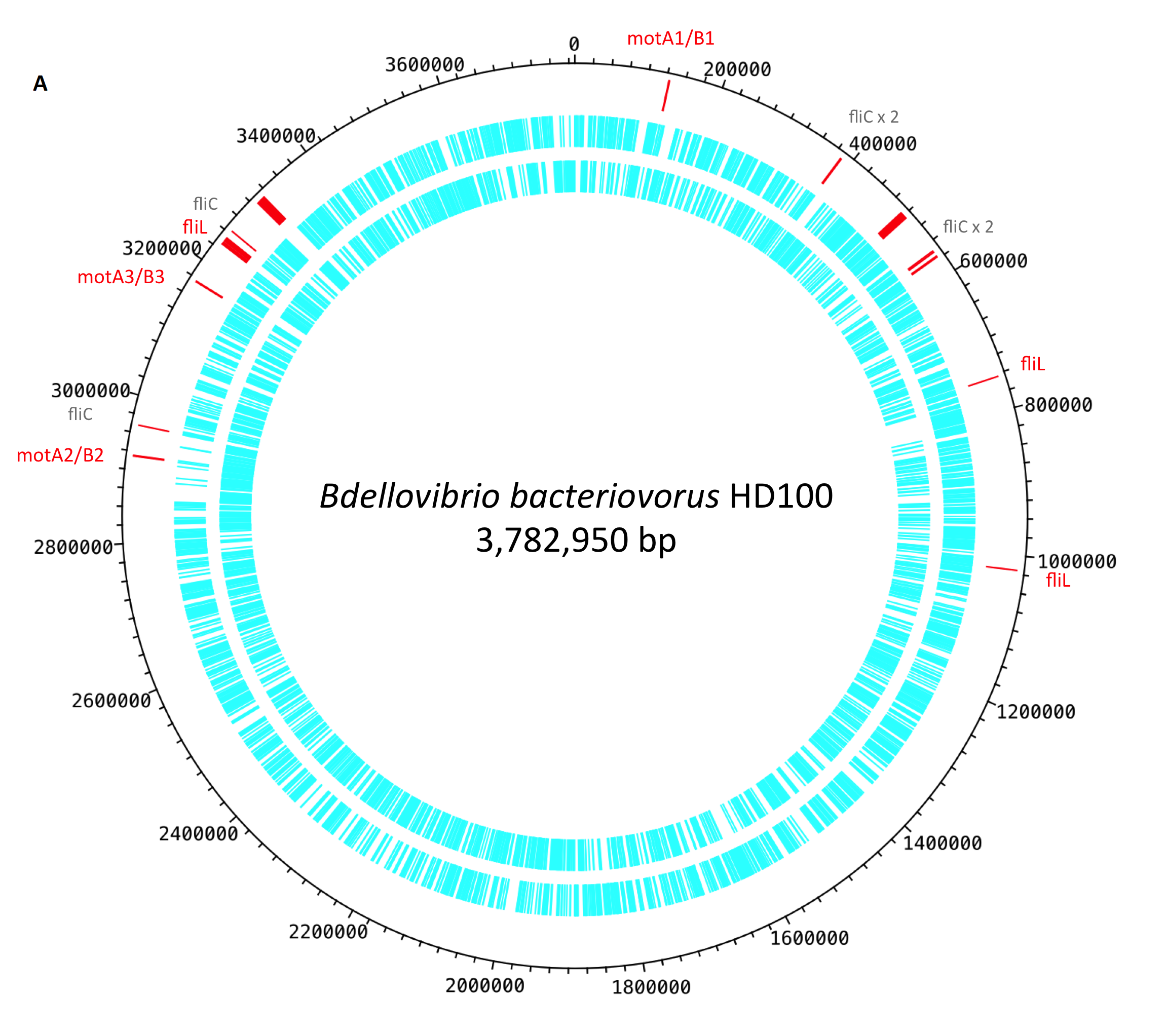


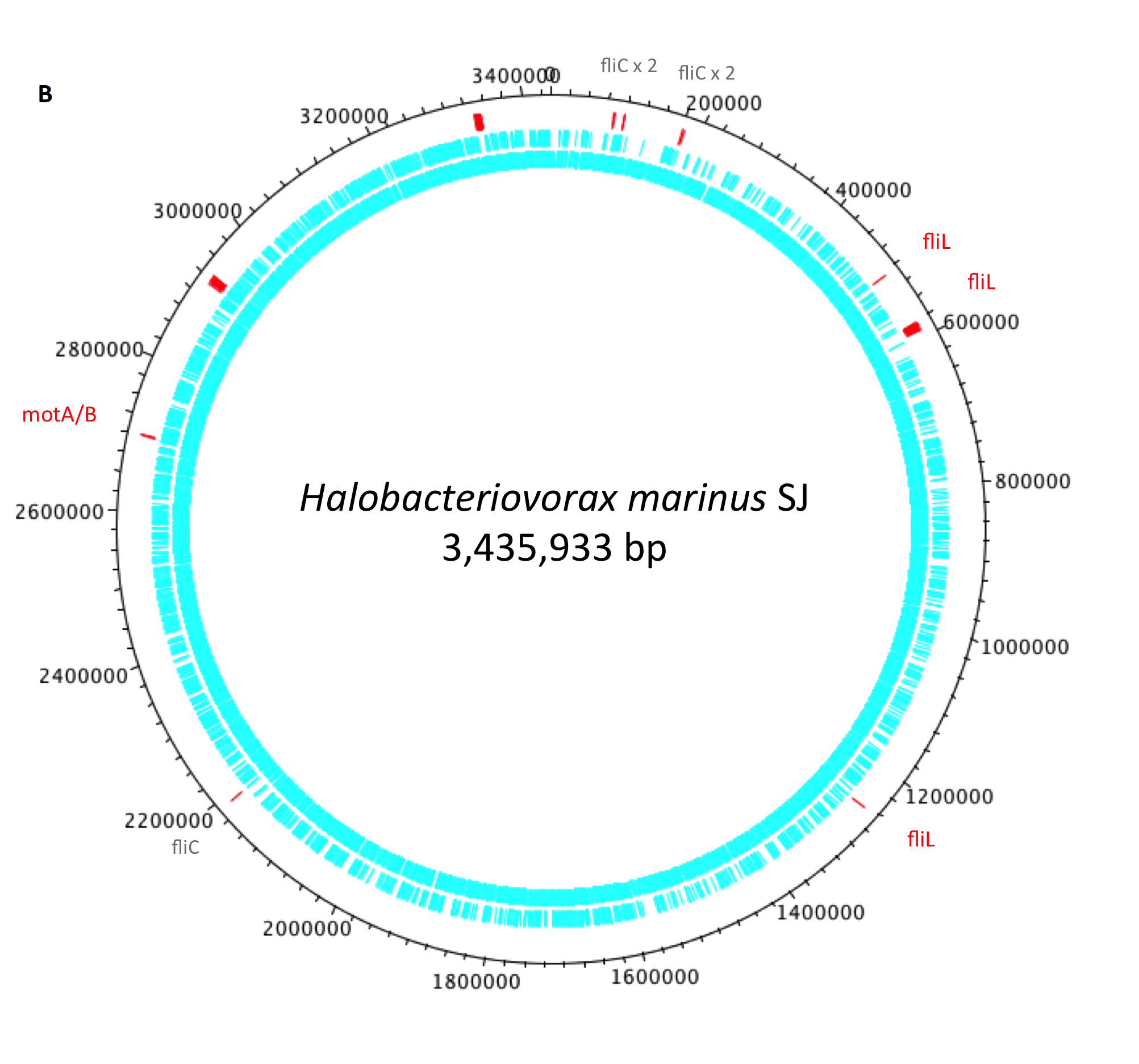


**Figure S15.** Flagellar genes clusters and their genomic organization in *Bdellovibrio* and *Halobacteriovorax.* Genome schemes of A. *Bdellovibrio bacteriovorous* H-D100 (GenBank entry NC_005363.1), and B. *Halobacteriovorax marinus* SJ (GenBank entry NC_016620.1). The outer circles show genome coordinates; the next level shows flagellar gene clusters including *fliL* or *motA/B* (red), followed by circles depicting protein coding genes on the forward then on the reverse strands (cyan). Details on the flagellar gene clusters are at table S5. Genome circles schemes were created from GenBank entries using the DNAPlotter program^4^.

**Figure S16**. A. Alignment of nucleotide sequences (268 n) of the N-terminal fragment of *bd1076*. C2/3/5/11/12bd1076MHI154 denote sequences of strains derived from the *bd1076* MHI154 mutant and complemented with the wild type allele from H-D100Sm. B. Alignment of nucleotide sequences of the upstream promoter region of *bd1076.* C1/2/5/8/10bd1076MHI167 denote sequences of strains derived from the *bd1076* MHI167 strain mutated in the gene promoter and complemented with the while type promoter of strain H-D100Sm.

**Figure S17.** Predicted protein-protein interactions network for FliL (Bd1076) using the STRING web tool (<https://string-db.org/cgi/about>). A. Scheme of the interactions network; B. Interaction scores, indicating the level confidence of the predictions, i.e., how likely an interaction is likely to be true, given the available evidence.

**Figure S18.** Gene expression of selected genes in SAS strains in different growth conditions along with one *bd1076* complemented MHI154 isolate. The two heat maps depict gene expressions of SAS MHI154 axenic (in PYE, planktonic and as biofilm), of SAS MHI167 axenic, and predatory MHI154 (attack phase (AP) cells in amHEPES) cultures, relative to parental H-D100Sm, in genes ascribed to various selected functions (A), and specifically, to genes encoding for stator components (B). Values were normalized to lon protease (*bd3749*) gene expression^97^. Gene expression is represented by fold change values in the heat maps. Within each row, cells with different letters are significantly different by Tukey’s test at 0.05% significance level. The significance level ‘A’ is assigned to the highest fold change value among treatments for any particular gene. A detailed result with statistical analysis can be found in Table S6. NE and NT represent no-expression and not tested, respectively. CdG: cyclic-di-GMP.

**Figure S19.** Cyclic di-GMP concentrations (ng/mg cell protein) in cultures from two independent experiments, in parental H-D100Sm (HD100SM) and H-I MHI154 (HI154) attack phase cells obtained from prey-dependent suspension in amHEPES buffer, and in MHI154 grown axenically in PYE. “HI154-PYE” are AP and elongating cells, and “HI154-PYE small” are filtered AP (small cells), free of elongating cells. Different letters above bars indicate differences by Tukey’s test at 0.05% significance level.


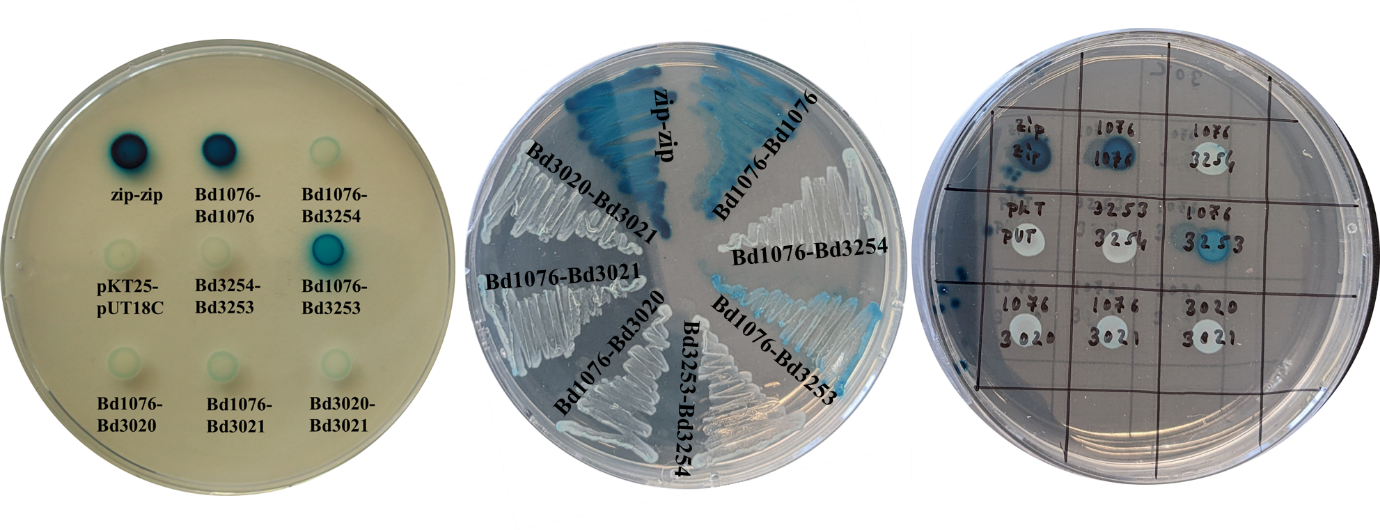


**Figure S20.** Bacterial 2-hybrid assays of flagellar stator proteins interacting with Bd1076. Couples of interacting proteins are indicated by the blue colonies, spotted on a LB plate containing kanamycin, ampicillin and X-gal after 48 hours of incubation at 28°C. Zip-zip, a positive control, represents interaction of T25-zip and T18-zip fusion proteins that can associate as a result of dimerization of the leucine zipper motifs appended to the T25 and T18 fragments of the adenylate cyclase, CyaA. pKT25-pUT18C is a negative control, showing that the two vectors used in the assay do not interact.


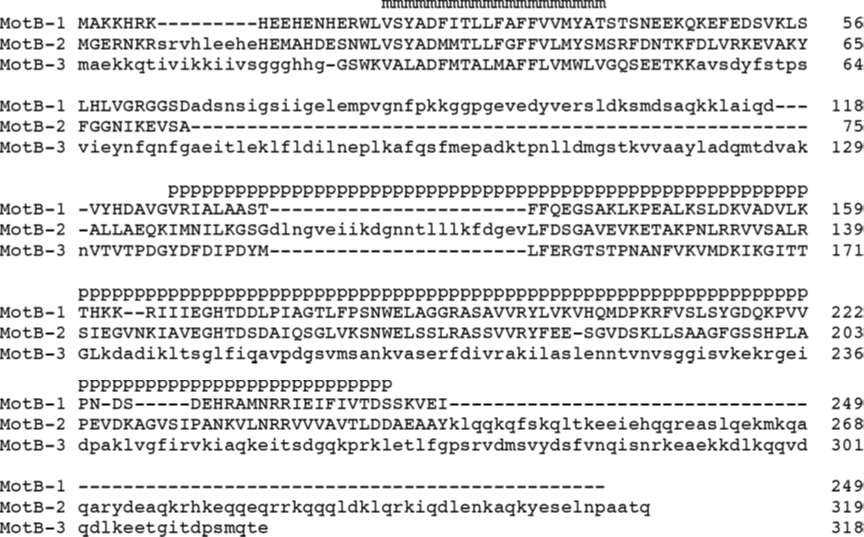


**Figure S21.** Multiple sequence alignment of *Bdellovibrio bacteriovorus* H-D100 three MotB proteins (Bd0145, Bd3020, Bd3253). Aligned residues are in upper case. “m” stretches above the alignment show probable transmembrane regions, and “p” stretches probable OmpA-type peptidoglycan-binding domains.

**References**

1. Cotter, T. & Thomashow, M. Identification of a Bdellovibrio bacteriovorus genetic locus, hit, associated with the host-independent phenotype. *J Bacteriol* **174**, 6018–6024 (1992).

2. Roschanski, N., Klages, S., Reinhardt, R., Linscheid, M. & Strauch, E. Identification of Genes Essential for Prey-Independent Growth of Bdellovibrio bacteriovorus HD100. *Journal of Bacteriology* **193**, 1745–1756 (2011).

3. Karunker, I., Rotem, O., Dori-Bachash, M., Jurkevitch, E. & Sorek, R. A Global Transcriptional Switch between the Attack and Growth Forms of Bdellovibrio bacteriovorus. *PLoS ONE* **8**, e61850 (2013).

4. Carver, T., Thomson, N., Bleasby, A., Berriman, M. & Parkhill, J. DNAPlotter: circular and linear interactive genome visualization. *Bioinformatics* **25**, 119–120 (2009).

5. Rotem, O. *et al.* Cell-cycle progress in obligate predatory bacteria is dependent upon sequential sensing of prey recognition and prey quality cues. *Proceedings of the National Academy of Sciences* **112**, E6028–E6037 (2015).
