# Supplementary material for: Flagellar stator genes control a trophic shift from obligate to facultative predation and biofilm formation in a bacterial predator
Source: mBio. 2024 Jul 22;15(8):e00715-24. doi: 10.1128/mbio.00715-24 (PMC11323537; doi:10.1128/mbio.00715-24)
Supplement: Supplemental text — Additional materials and methods. [file mbio.00715-24-s0002.docx]

**Supplementary materials and methods**

**Bacterial strains, media, and growth conditions.**

*Bdellovibrio bacteriovorus* H-D100Sm (a kind gift from Eckhard Strauch) and *Escherichia coli* ML35 were used as predator and prey, respectively. Prey was grown overnight in Luria-Bertani (LB; Difco Laboratories, USA) at 37^o^C on a rotary shaker at 180 rpm. Overnight cultures were harvested and concentrated to a final optical density (OD_600_) of 10 corresponding to 10^10^ CFU ml^−1^. Starter predatory cultures were prepared at 28°C by co-culturing the predator with *E. coli* ML35 (starting OD_600_ = 0.2) in amended HEPES (amHEPES) buffer containing 50 µg/ml streptomycin (Sm), 3 mM MgCl_2_•6H_2_O and 2 mM CaCl_2_•2H_2_O. Host-Independent (H-I) strains were grown in similarly amended peptone yeast extract (amPYE, pH 6.8), and when required, prey extract (see below).

**Environmental effects on H-I biofilm development.**

The effect of temperature (17, 22, 25, 30, 35 and 40°C), pH (5, 6, 7, 8 and 9), and divalent cations (3 mM MgCl_2_•6H_2_O and 2 mM CaCl_2_•2H_2_O) on the formation of H-I strains (representatives from BFF, SAD and SAS) biofilms was measured, using the CV assay.

**Characterization of H-I strain biofilm extracellular polymeric substances (EPS).**

Biofilms were formed in microtiter plates using the MHI179*-tdt* strain, including three rinses to remove planktonic cells. They were treated with different enzymes at different concentrations and subsequently monitored for EPS removal. Proteinase K (≥3.0 unit/mg, Sigma), bromelain (≥3 units/mg protein, Sigma), deoxyribonuclease I (≥2,000 KU/mg protein, Sigma), α-amylase (~30 U/mg protein, Sigma) and lyticase (≥500 U/mL, Sigma-Aldrich) were individually added to biofilm-containing wells and incubated for 2 h at 37°C in the presence of sodium azide (50mM) to avoid bacterial growth. Pre-formed biofilm reduction was calculated after CV staining as the mean of removal (in %) by each enzymatic treatment, compared to the untreated biofilm.

Extracellular DNA and proteins were differentially stained to characterize the distribution of these components in the biofilm matrix. MHI179-*tdt* biofilms were developed on 8 well µ-Slides as above. Proteins were stained using fluorescein isothiocyanate (FITC) isomer I (46.6 μg/ml, Sigma-Aldrich). Extracellular DNA was stained with 100 μM DiTO™-1 (AAT Bioquest). Stained slides were incubated in the dark at RT for 30 min, subsequently washed thrice with amHEPES and visualized under epifluorescence microscopy as above.

**Predation by H-I strains**

To monitor predation by H-I strains, the *tdt*-carrying pMQ414 plasmid was introduced into the parental H-D100Sm strain, and into BFF MHI179, surface associated (SAS) MHI154 and surface adhering (SAD) MHI153 strains by conjugation as in^1^. Transformed predator and prey strains were mixed at a ratio of 10^7^:10^9^, respectively, in black μclear sterile lid plates (Greiner Bio-One GmbH, Austria) to a final volume of 200 μl per well and incubated in a plate reader at 28°C with shaking every 30 min at 810 rpm, for up to 48 hours. OD_600_ and fluorescence (excitation at 550 nm, emission at 590 nm) were automatically recorded every 20 min. Axenic prey and predator cultures were used as controls. Predator and prey counts were performed at the culture end-point time by dilution plating using double-layered agar and LB agar^2^ , respectively. Effect of temperature on predation by the obligate H-D parent and H-I predators was measured in 1 ml culture tubes monitored manually through OD measurement. Predation on *Pseudomonas fluorescens* biofilm was performed as in^3^: A *P. fluorescen*s biofilm was grown for one day in microtiter wells, then exposed to 10^7^ cells ml^−1^ of the parental H-D100Sm, SAD MHI153, SAS MHI54 or SAS MHI167 strains. Biofilm-associated prey and predator populations were measured by dilution plating using double-layered agar and LB agar after discarding planktonic cells, and subsequently dislodging biofilm-associated cells in amDDNB (a 1:50 dilution of nutrient broth with 3 mM MgCl_2_•6H_2_O and 2 mM CaCl_2_•2H_2_O) through vigorous pipetting.

**Scanning electron microscopy (SEM).**

Biofilms formed by the MHI179, MHI154 and MHI153 strains were examined by SEM following^4^. In brief, biofilm structures were developed on glass coverslips for 2 days, and fixed using 2.5% glutaraldehyde in cacodylate buffer (0.1 M) for 1 hr. Coverslips were washed to remove the fixative and serially dehydrated using a gradient ethanol series (25, 50, 75, 95, and 100%). Finally, samples were fully dried in a Critical Point Dryer (Quorum K-850) and gold-coated (Quorum Q150T ES). The samples were observed under a scanning electron microscope JEOL model JSM-7800f.

**Epifluorescence and confocal microscopy.**

Biofilm formed by the Tdt protein-expressing MHI179-*tdt*, MHI154-*tdt* and MHI153-*tdt* strains on a µ-Slide 8 well (ibidi) were visualized under epifluorescence using an inverted microscope and an RFP filter (Nikon TiE, Japan). Images were acquired and processed with a NIS AR elements software.

Confocal microscopy was used to obtain 3D data. Biofilms developed in a µ-Slide 8 well slide were washed to remove planktonic cells and directly observed under confocal microscopy (Leica SP8 Lightning Confocal). Biofilm structure was analyzed by taking a series of horizontal sections (at 0.5 µm intervals). Subsequently, images (>10 per sample) were analyzed by ImageJ, to obtain the fraction of surface covered by the biofilm.

**PCR and gene sequencing.**

The primers used in this study, and amplification conditions are detailed in Table S1. Variations in the *bd0108* gene were analyzed by high-throughput sequencing. *bd0108* amplicons were obtained from pools of H-I strains Type-I, Type-II, SAS and BFF. DNA was extracted from the four pools and PCR performed using the DSBD0108 primer set including a universal CS1/CS2 linker sequence (Table S1). The samples were sequenced pair-ends on an Illumina MiSeq (Research Resources Center, University of Illinois Chicago) platform with a read length of 2x301 bp, yielding 57,669 ± 1,809 paired-reads per sample.

Raw fastq files were initially subjected to quality control and primer trimming using BBDuk tools (https://jgi.doe.gov/data-and-tools/bbtools). Further de-noising was done using dada2^5^, totalling 171,994 reads from four samples. Forward and reverse reads were truncated at the 260, and 170 base positions, respectively. Paired reads were processed and merged after strict quality control (maxN=0, maxEE=c(2,2), truncQ=3). 109,695 merged, non-chimeric reads were grouped into amplicon sequence variants (ASVs) according to 100% sequence similarity.

**RT-qPCR.**

The STRING webtool (<https://string-db.org/>) was used to predict proteins with a potential to interact with FliL Bd1076, and primers were designed for the predicted genes. RNA extraction and cDNA synthesis were carried out as in^6^. Total RNA was extracted using TRI Reagent (Sigma-Aldrich, USA) then purified using Direct-zol RNA purification kit (Zymo Research, USA). DNA contamination was removed by DNase I (NEB, USA) treatment, with a successive purification step using an RNA Cleanup Kit (NEB, USA). Complete genomic DNA removal was confirmed by 16S *rRNA* amplification using *Bdellovibrio* specific primer set 16SRNAex (Table S1). An equal amount of total RNA was used for cDNA synthesis with each sample (TIANGEN Biotech, Beijing). Specific primers designed for representative genes were used to amplify cDNAs (Table S1) and reactions were performed in a 96-well plate (Applied Biosystems) with MicroAmp® Optical Adhesive Film (Applied Biosystems) in a Step One plus Real-time PCR System (Applied Biosystems). Each 10 μl reaction consisted of 5 μl of SYBR® Green PCR Master Mix (Applied Biosystems). 0.1 μl of each primer, 0.5 μl of cDNA, completed with PCR grade DDW. Thermal cycling was performed using a standard program mode, followed by a melt curve (Table S1). Relative gene expression is presented as the fold change of target gene expression in the tested isolates relative to expression in H-D100Sm AP cells normalized to the Lon protease reference gene (*bd3749*)^7^ with 2^−ΔΔCT 7^.All gene expression analyses were repeated at least twice with triplicate measurements.

**Whole genome sequencing.**

Whole-genome sequencing was performed using the Novaseq platform (Research Resources Center, University of Illinois Chicago) with a read length of 2x150 bp. Sequencing data for the obligate H-D100Sm parental strain and for 7 derived H-I strains were assembled individually using the Spades sequence assembler (v3.14.1) on the Illumina raw data. Following the first assembly round, the spades parameters were modified (adding a k-mer length of 99 to the recommended 21, 33, 55 & 77). This generated a lower number of scaffolds, including long (>1Kb) and high coverage (>180x) ones for each of the 8 strains, resulting in 2- 3 such scaffolds and a 193 bp high coverage scaffold for each. The assembly of H-D100Sm parental strain was compared with a consensus of the H-I strains derived from it, and with the H-D100 strain parent of H-D-100Sm. Differences between the sequenced genomes themselves, and with other related genomes were found using BLAST sequence alignment methods^8^.

FliL proteins were identified by searching the GenBank-predicted proteomes of the genomes listed in table S5 using the psi-blast program^9^ with a multiple sequence alignment of pfam (Mistry *et al.*, 2021) entry PF03748.17 seed alignment as query, a single iteration, and e-value threshold of 1e^-3^ or less. The resulting proteins were then all used as tblastn search queries against the genome sequences to verify all *fliL* genes were translated in the proteomes. MotA and MotB proteins were identified by searching the GenBank-predicted proteomes of the genomes listed in table S5 using the blastp program^9^ with the *Bdellovibrio bacteriovorus* H-D100 and *Halobacteriovorax marinus* SJ MotA and MotB proteins as queries, with an e-value threshold of 1e^-6^ or less. The resulting proteins were then all used as tblastn search queries against the genome sequences to verify all MotA and MotB genes were translated in the proteomes. This procedure identified all MotA and MotB proteins, together with other MotB-like proteins (e.g. OmpA family proteins). Genuine MotB proteins were verified as such by their genomic positions immediately downstream of each gene coding for an identified MotA protein.

**DNA manipulations.**

A *bd1076* (*fliL*) marker-less deletion was generated^10^. Spliced overlap extension PCR was used to join 1 kb flanking regions on either side of *bd1076*; the DNA products were then inserted into suicide vector pK18mobsacB through restriction-free (RF) cloning for multicomponent assembly^11^ and conjugated into the parental H-D100Sm, BFF MHI39 and BFF MHI179 strains using the conjugative donor strain *E. coli* S17^10^. The subsequent merodiploid *B. bacteriovorus* isolates, where the plasmid was incorporated into the genome via a single cross-over in the flanking regions of *bd1076*, was then grown with sucrose supplementation to induce a second recombination event to remove the target *bd1076*, leaving only the flanking regions. Similarly, chromosomal complementation of *bd1076* was performed using a PCR product containing the wild-type allele along with 500 bp from the flanking regions on either side, cloned into pK18*mobsacB* and introduced into the mutant strain as above. Details of the primers used are in Table S1. All engineered strains were confirmed through sequencing and *bd1076* expression analysis.

Chromosomal complementation of *bd0108* was performed using a PCR product containing the type strain allele (isolated from *B. bacteriovorous* H-D100) along with 300 bp from the flanking regions on either side, cloned into pK18*mobsacB* and introduced into the mutant H-I strain, i.e., MHI39. Primers pK18bd0108 and pK18bd0108-Rev used for restriction free cloning (Table S1). The engineered strains were confirmed through sequencing.

Biofilm formation of the complemented strains were tested by crystal violet staining followed by OD measurement at 600 nm.

**CdG concentrations**

Strains H-D100Sm and MHI154 grown with prey in amHEPES, and axenically in PYE for MHI154, each in 24 mL were two-fold concentrated by centrifugation followed by the addition of 12 mL 20 mM Tris-HCl (pH 8.0). Cells were then disrupted by sonication at 70% amplitude, for 17 sec ON/20 sec OFF for a total of 18 min (Qsonica). Cells debris were removed by centrifugation, the supernatant was utilized for detecting c-di-GMP using the Cyclic di-GMP ELISA Kit (Cayman). The obtained values were normalized by the protein concentration of the supernatant at 3620 g for 20 min, at 4 °C, and the supernatant pipetted in c-di-GMP-detection microtiter plates in two biological replicates with two technical replicates, using the Cyclic di-GMP ELISA Kit (Cayman) according to manufacturer’s instructions.

Strains H-D100Sm and MHI154 grown with prey (24 mL) in amHEPES, and axenically in PYE for MHI154 (axenic) (24 mL) were concentrated 2-fold to a final volume of 12 mL in 20 mM Tris-HCl (pH 8.0), and disrupted by sonication at 70% amplitude, 17 sec ON/20 sec OFF; 18 min total sonication time (Qsonica). After centrifugation (3620 g for 20 min at 4 °C), the supernatant was utilized for detecting c-di-GMP using the Cyclic di-GMP ELISA Kit (Cayman), in two biological replicates with two technical replicates. Optical density was measured at 450 nm, and values were plotted to determine the sample c-di-GMP concentration. The results were normalized against protein concentration using the Bradford method^12^.

**Statistical analysis**

Statistical analysis was performed using the JMP Pro (version 16) and GraphPad Prism program (version 9). All the data were subjected to one-way ANOVA with Tukey’s multiple comparison test. P values are represented by GraphPad style i.e., 0.1234 (ns), 0.0332 (*), 0.0021 (**), 0.0002 (***), <0.0001 (****).

**References**
