# Supplementary material for: Flagellar stator genes control a trophic shift from obligate to facultative predation and biofilm formation in a bacterial predator
Source: mBio. 2024 Jul 22;15(8):e00715-24. doi: 10.1128/mbio.00715-24 (PMC11323537; doi:10.1128/mbio.00715-24)
Supplement: Supplemental tables — Tables S1 to S6. [file mbio.00715-24-s0003.pdf]

Table S1: Primers and PCR conditions used in this study.

Primer set	Primer	Sequence (5'-3')	Description	Product size (bp)	Annealing temperature (˚C)	Time of elongation	Number of cycles	Screened isolates
hit	3F	TAGACAGATGGGATTACT	Bdellovibrio's hit region specific primer	~900 bp	50	1 min	30	
	913R	GTGTGATGACGACTGTGA-3′						
Bd0108	BD0108minus102F	TATTGCATCGCGTGTGCCAG	Primer specific to the upstream and downstream of BD0108 gene	563	53	1 min	30	MHI 39, 92, 154, 167 and 179
	BD0108down133R	CAACAAGGTGGACAGCAAAGA						
RhlB	RHLBminus135F	GAAGCCTCCAACCAAGACACG	Primer set to amplify three overlapping fragments containing full RHLB gene	661	52	70 sec	30	MHI 39, 92, 154, 167 and 179
	RHLB526R	GGGTCGCATTGAAGATTATCTGAA						
	RHLB355F	AGGACCTCTGTCACCACCAC		774	56	85 sec	30	
	RHLB1128R	GCGGACAGAATGTTTGATATGGGC						
	RHLB921F	CGTGGAAGATCTGGTCTTTCA		860	51	90 sec	30	
	RHLBdown130R	GGGTCCACTCAGATCAACCT						
PcnB	PCNB530F	GACCCGAAGGCGCGTTTCAT	Primer set to amplify two overlapping fragments containing full RHLB gene	791	56	85 sec	30	MHI 39, 92, 154, 167 and 179
	PCNBdown112R	AAGACAATCCACGCTGGGTGA						
	PCNBminus105F	GAGGGTGCCGTCATGTTGAT		832	55	90 sec	30	
	PCNB667R	CGCAGGAACTTCAGCCACTC						
Bd2981 (rpsL)	rpsLF	AGTCTTGACCTGGGCGTCA	Primer specific to the upstream and downstream of rpsL gene	612	55	70 sec	30	MHI 39, 154, 167 and 179
	rpsLR	CAAGCTCTTTCAAAGCACCGTAGA						
dgcC	dgcC116minusF	TTCCCATCAGACACTCCTTTG	Primer set to amplify two	872	53	90 sec	30	

	dgcC756R	ATAGCTTCTGCGGTTGTGAG	overlapping fragments containing full dgcC gene					MHI 39, 154, 167 and 179
	dgcC641F	CAAACGTATGGAGGCGATCAA		714	53	80 sec	30	
	dgcC128downR	TGTATCTGAACCGGAAATTCACTG						
pilB	pilBminus133	GTCTGCGTCCGGATCATAAT	Primer set to amplify three overlapping fragments containing full pilB gene	752	51	80 sec	30	MHI 39, 154, 167 and 179
	pilB619	CGTAAGGTTCAAAGTGAATATCCG						
	pilB548	TCTGAAGACGCGCCGATCAT		741	56	80 Sec	30	
	pilB1269	AGGTGCATCGTTGGTATGCAG						
	pilB1192	GGGGAGATTCGTGACCTTG		661	54	70 sec	30	
	pilBdown132	GTCATCAGCGCTGGCGTT						
fliLup	FliLinterver_F	GCAGACTCAACGCAAATAAAG	Sequence analysis of full fliL (BD1076) gene and upstream promoter	~776	49	75 sec	30	MHI133, MHI137, MHI228, MHI315, MHI129, MHI155, MHI139, MHI305, MHI355, MHI167 and MHI71
	FliLinterver_R	GGGCCATGATTACAACGAA						
fliLdown	FliLinterverNew_F	CAGCCCGTGAGCAATAGTC	Sequence analysis of full fliL (BD1076) gene and downstream region	799	51	75 sec	30	MHI154
	FliLinterverNew_R	ATGCTTCTTTCGTTGGTTATGC						
motA	motAverF	CCCTCTGGAGCTGATGATTA	Sequence analysis of full motA (BD3254) gene	648	51	70 sec	30	
	motAverR	ACCATCGCAGCTTTGATAC						
	Pk18_upfliL_F	cactggccgtcgttttacaacgtcgtgactCAGATTC ATAGCGCTGCAGGG	Contruction of fliL (BD1076)	1060 bp	68	2 min	30	

pK18*mobsacB*:Δ*fliL*	upfiL_downfliL_R	TAAGACTTTTGGCAGATCTTGCGACAGGAATTATAACCCTCTGTTCATTGAAACTATCGGTG	knockout clone pK18*mobsacB*:Δ*fliL*					
	upfiL_downfliL_F	CCGATAGTTTCAATGAACAGAGGGTTATAATTCCTGTCGCAAGATCTGCC		1060 bp	64	2 min	30	
	downfliL_pk18_R	ctcttcgctattacgccagctggcgaaaggCTTATAGGCAACTACGAAAGAAGTG						
Δ*fliL* mero	FliLupMero_F	GGACTTGAGGGCATAGATTG	Δ*fliL* merodiploid verification,	2596 bp and 2089 bp	49	2 min	30	
	FliLupMero_R	CAGATCCAGAGCGTACATATC						
Δ*fliL* knock out	FliLKOVerF	TTTGTCCACCAGAATCACATAG	Δ*fliL* knock out confirmation	592 bp for positive kockout	50	1.30 min	30	
	FliLKOVerR	CGGCAGGGAAATCTTCAAA						
pK18*mobsacB:fliL*	Pk18_fliLcomp_F	cactggccgtcgttttacaacgtcgtgactGGAATTTTTGGCTGGCTCAGAC	Contruction of fliL (BD1076) complementation clone pK18*mobsacB*:*fliL*	1507 bp	68	1.30 min	30	
	downfliL_pk18Comp_R	ctcttcgctattacgccagctggcgaaaggTGGTGGTGAAAGCATTGTTGGTG						
pK18 *mobsacB* RF Val	pK18RFVal_F	atcctctagagtcgacctg	Validation of positive RF clone in pK18*mobsacB*	150 bp without insert	50	1.30 min	30	
	pK18RFVal_R	ctgcgcaactgttgggaa						
pK18*bd0108*-comp	pK18*bd0108*-Fw	CGTCGTTTTACAACGTCGTGACTGTATCCTATAAACCAAATGGTGAAA	Complementation of mutated *bd0108* in MHI39	606 bp	55	25 sec	30	
	pK18*bd0108*-Rev	TATTACGCCAGCTGGCGAAAGGAAGAACTCAATGAAGGCTAT TGCA						
FliLex	fliLexF	GTGATGGGTGCTGGTGGTTA	fliL expression analysi using primer set designed from 5' ends	119	60	25 sec	30	
	fliLexR	GCCAGGTCCGTTTCATCAGA						
FliLendEx	FliLendExF	GAGGTGATGGAGCCGGAAAA	fliL expression analysi using primer set designed from 3' ends	168	60	25 sec	30	
	FliLendExR	AACATCTTTCACGACGCCAC						
16SRNAex	16SRNAexF	CGC ATA AGA CCA CAG GAG CT	*B. bacteriovorus*'s	114	60	25 sec	30	
	16SRNAexR	AAA GAT CGT CGC CTT GGT GA						

			16S *rRNA* specific					
Bd0532	BD0532nF	AGTGTGGAGATTTCCCAGCG	Bd0532 (FlgA), flagellar basal-body P-ring formation protein	101	60	25 sec	30	
	BD0532nR	AGAACCACAGCATCCAGAGC						
Bd3328	BD3328F	CTTCCGCGTTTCTTTGTCATC	Bd3328 (FliM), Flagellar motor switch protein	107	60	25 sec	30	
	BD3328R	TCGGCATTGGCAGTGTATT						
Bd1075	BD1075F	ACGATCAGTTGCTGGTGAAA	Bd1075; L,D-transpeptidase	90				
	BD1075R	CGGACTTGAGGGCATAGATTG						
Bd0536	BD0536F	TGCGCGAAGTTTCTGATATGTA	Bd0536(FlgJ), Flagellar protein peptidoglycan hydrolase	105	60	25 sec	30	
	BD0536R	CAGCCTGATTGGACTGAATGA						
Bd3407	BD3407F	GTCAGAGAACGCGATCATGTA	Bd3407 (FlgB), Flagellar basal-body rod protein	82	60	25 sec	30	
	BD3407R	TTCGGTTGCGGCATACTT						
Bd3404	BD3404F	CTGACGACGCTGGAAAGAAA	Bd3404(fliF), Flagellar M-ring protein	95	60	25 sec	30	
	BD3404R	CGCACCACGATGAAGAAGAA						
Bd3398	BD3398F	AAGCCATCGAGTCCAGTCTA	Bd3398 (FliK), Flagellar hook-length control protein	90	60	25 sec	30	
	BD3398R	AGTTCACCACATCCACCTTTAC						
Bd3329	BD3329exF	ATATGCTCGTCGTTGCAGGT	Bd3329 (FliL), Flagellar basal body-associated protein	125	60	25 sec	30	
	BD3329exR	TTCAGTGGCCTCTTTGTGCT						
Bd0804	BD0804exF	CCGTGATGCGCTTAAGTTGG	Bd0804 (FliL), Flagellar basal body-associated protein	106	60	25 sec	30	
	BD0804exR	AAGCCCTGGGCAGTTCTTTT						
Bd3253	BD3253F	TGGCGTTCTTCCTGGTTATG	Bd3253(motB), motor protein	85	60	25 sec	30	
	BD3253R	ACAGAAGGTGTGGAGAAGTAATC						
Bd3021	BD3021exF	CAATCCTCGATGCGGCAAAG	Bd3021(motA), motor protein	164	60	25 sec	30	
	BD3021exR	GACTCTTTCACGACCCAGCA						
Bd3020	BD3020exF	TCGATGTCCCGCTTTGACAA	Bd3020 (motB), motor protein	109	60	25 sec	30	
	BD3020exR	TTTGTTCAGCCAGCAATGCC						
Bd0144	BD0144exF	TAAAGACCGGTCTGCAGCTG	Bd0144(motA), motor protein	145	60	25 sec	30	
	BD0144exR	TCGATACGGTTTAGACGCGG						

Bd0145	BD0145exF	TTCCCTAAAAAGGGCGGTCC	Bd0145 (motB), motor protein	151	60	25 sec	30	
	BD0145exR	AGAATGTCGAAGCGGCAAGA						
Bd0367	BD0367F	CCACAAGCTCATCGAATCCT	Bd0367(dgcA), Two-component response regulator with GGDEF domain	97	60	25 sec	30	
	BD0367R	CTCACGCACCGACTTCAATA						
Bd1434	BD1434F	GCATCAAAGCACCCACAAC	Bd1434 (dgcC), GGDEF domain protein	99	60	25 sec	30	
	BD1434R	GTGGTGTTGGCCTCCATAA						
Bd0742	BD0742F	GAGTTTGTGCTGCTGCTTTC	Bd0742 (dgcB), GGDEF domain protein	114	60	25 sec	30	
	BD0742R	CGGGATCTTCTTGCCTTCAA						
Bd3125	BD3125nF	AAGACTGAAAGAGAGCGCCC	Bd3125 (cdgA), Two-component system sensor histidine kinase	126	60	25 sec	30	
	BD3125nR	GAGCTGACCGCAATAAAGCG						
Bd2503	BD2503F	AATCGCCCTGCCTATTCTTAC	Bd2503 (mcp), Methyl-accepting chemotaxis sensor/transducer protein	124	60	25 sec	30	
	BD2503R	CTCTGCTGACTTTGTGACCTTA						
Bd1126	BD1126F	GACAAGGCCCTGGATGAAA	Bd1126 (mcp), Methyl-accepting chemotaxis sensor/transducer protein	97	60	25 sec	30	
	BD1126R	GAATCTGGAAGGTACTGGAGATG						
Bd0932	BD0932F	GTGCTTTCATGTCCGCTATTC	Bd0932 (mcp), Methyl-accepting chemotaxis sensor/transducer protein	98	60	25 sec	30	
	BD0932R	CACTCCGTCGGTAACTTTCT						
Bd2406	BD2406F	CCATTGAAGCTCTGGACAATCT	Bd2406 (CheA), Chemotaxis protein histidine kinase and related kinases	101	60	25 sec	30	
	BD2406R	GATCTGCACCTTGCCTTCTT						
merRNA	merRNAexF	GTGCTAGCGAGGAATCGTCA	massively expressed riboswitch RNA	161	60	25 sec	30	
	merRNAexR	AAGCTAAAGGGGTGTCGGTG						
Bd3734	Bd3734exF	ATCCAAGCTGGTGGCATTGA		122	60	25 sec	30	

	Bd3734exR	GACCTGGAACGGTGTACAGG	Bd3734 (MglA), mutual gliding-motility protein						
Bd2761	Bd2761exF	CACATCGAGATGGATGGCGA	Bd2761 (RomR), essential for viability in both predatory and non-predatory modes	103	60		25 sec	30	
	Bd2761exR	AAATGAAGGAGTCCGCAGCA							
Bd2492	Bd2492exF	GAACGAACACAAGCAGCGTT	Bd2492 (TPR), predicted tetratricopeptide repeat (TPR) domains typically involved in protein-protein interactions	113	60		25 sec	30	
	Bd2492exR	GAGCCTGTTCTGTTTCCGGA							
Bd2494	Bd2494exF	ACGCTTTCACCTTGAAACGC	TamAB-like transport activity might be required for OMP/autotransporter proteins involved in predation	173	60		25 sec	30	
	Bd2494exR	GGTCACACCCACGTTCAGAT							
Bd2495	BD2495exF	TGGGACAAATCGAAGAGGCC	TamAB-like transport activity might be required for OMP/autotransporter proteins involved in predation	133	60		25 sec	30	
	BD2495exR	TTCAGGGTGGCTTTGGTGTT							
Bd0760	BD0760exF	TCGATATTACGCCACGTCTAAG	Flagellar brake protein	89	60		25 sec	30	
	BD0760exR	GGCCTCTGTAATGTCGAGAAC							
Bd1007	BD1007nexF	TCTGGGCGAAGTCGTGAAAA	Flagellar brake protein	184	60		25 sec	30	
	BD1007nexR	TTCTTTGAATTTGCGCGCCA							
DSBD0108	DSBD0108CS1F	ACACTGACGACATGGTTCTACACAGGTAGCCAGCACATTTAATAAC	Deep sequencies of BD0108 gene	457	51		25 sec	30	

	DSBD0108CS1F	TACGGTAGCAGAGACTTGGTCTGTCAGGTTTCTAGACAGATGGG	from H-I consortium					
pKT25:Bd1076	pKTBd1076_F	CAGGGTCGACTCTAGAGGATCCCCGGGTAATGGCTGAAGCACAAGAGAC	Construction of pKT25:Bd1076	567	65	60 sec	30	
	pKTBd1076_R	ACGACGGCCAGTGAATTCTTACTTACTTAGGCTACTGAACGACGAAATCAGAGA						
pKT25:Bd3021	Bd3021_pKT25F	CAGGGTCGACTCTAGAGGATCCCCGGGTAATGAATATAGCGGGGCTTTTAGGT	Construction of pKT25:Bd3021	875	67	70 sec	30	
	Bd3021_pKT25R	GTAAAACGACGGCCAGTGAATTCTTACTTACTTAGGTTACGCCGCCTTCTTCGCT						
pKT25:Bd3253	pKTBd3253_F	CAGGGTCGACTCTAGAGGATCCCCGGGTAATGGCAGAGAAGAAACAGACG	Construction of pKT25:Bd3253	1017	65	90 sec	30	
	pKTBd3253_R	ACGACGGCCAGTGAATTCTTACTTACTTAGGTTATTCTGTCTGCATCGACGG						
pKT25	pKTRFverF	ATCGACATGTTCGCCATTAT	Verification of pKT25 constructs	223 (without insert)	50	60 sec	30	
	pKTRFverR	AGGCGATTAAGTTGGGTAAC						
pUT18C:Bd1076	pUTBd1076_F	GCCACTGCAGGTCGACTCTAGAGGATCCCATGGCTGAAGCACAAGAGAC	Construction of pUT18C:Bd1076	566	65	60 sec	30	
	pUTBd1076_R	TTATATCGATGAATTCGAGCTCGGTACCCGCTACTGAACGACGAAATCAGAGA						

pUT18C: Bd3254	pUTBd3254_F	GCCACTGCAGGTCGACTCTAGAGGATCCCATGGGTTTTGTAGGTATATTAGTTGT	Construction of pUT18C:Bd3254	927	62	80 sec	30	
	pUTBd3254_R	GTTATATCGATGAATTCGAGCTCGGTACCCGTTAAGCTGCTTTCTTAATTTCTTTCG						
pUT18C: Bd3020	Bd3020_pUT18CF	GCCACTGCAGGTCGACTCTAGAGGATCCCATGGGAGAGCGTAATAAACGC	Construction of pUT18C:Bd3020	1020	66	90 sec	30	
	Bd3020_pUT18CR	GTTATATCGATGAATTCGAGCTCGGTACCCGCTACTGAGTCGCCGCAGGA						
pUT18C	pUTRFVerF	AAAGCCTGTTCGACGATGG	Verification of pUT18C constructs	235 (without insert)	52	60 sec	30	
	pUTRFVerR	CGGCATCAGAGCAGATTGTA						
Bd0081	BD0081exF	GCTGCAAACAGCTTTGTCGA	adenylate cyclase which putatively binds to c-di-GMP	152	60	25 sec	30	
	BD0081exR	TACTGGCAAGCTTTGAGCGA						
Bd1971	BD1971exF	CTGGGGGAAGGCGAAATCTT	cyclic-di-GMP hydrolase carries a CRP domain	144	60	25 sec	30	
	BD1971exR	GACTTTGTCAGCGGTGGAGA						
Bd2590	BD2590exF	CCCTCAGCCCAACCTTGAAT	coding for a Clp-like c-di-GMP binder is cotranscribed with *bd2591*, coding for a standard cyclic AMP receptor protein	132	60	25 sec	30	
	BD2590exR	ATGATCTGGCCGGCTTTGAA						

Table S2: List of randomly selected and verified different biofilm-formers categorized here as Surface associated (SAS), Surface Adherers (SAD) and Biofilm Formers (BFF).

**SAS**	**SAD**	**BFF**
MHI71, MHI121, MHI129, MHI133, MHI137, MHI139, MHI142, MHI143, MHI144, MHI151, MHI152, MHI154, MHI155, MHI156, MHI157, MHI167, MHI174, MHI184, MHI206, MHI227, MHI228, MHI301, MHI305, MHI355	MHI40, MHI53, MHI153, MHI171, MHI173, MHI191, MHI192, MHI194, MHI195, MHI219, MHI221, MHI223, MHI226, MHI307, MHI308, MHI311, MHI312, MHI313, MHI314, MHI315, MHI316, MHI317, MHI318, MHI319, MHI353	MHI39, MHI47, MHI67, MHI84, MHI87, MHI88, MHI89, MHI92, MHI93, MHI159, MHI176, MHI179, MHI205, MHI218, MHI230

Table S3: Percentage of mutated *bd0108* alleles in host-independent strains.

	**BFF**	**SAS**	**Type-I**	**Type-II**
Random sequencing	82% (9/11)	5% (1/19)	NA	32% (14/44)
Consortium sequencing	23%	32%	25%	21%

NA= not analysed

Table S4: Number of *fliL* genes and *motA, motB* gene clusters in *Bdellovibrio*- and *Bacteriovorax*-like species with fully determined genomes

**Species** [a]	**fliL**	**motA/B-clusters**
Bdellovibrio bacteriovorus HD100	3	3
Bdellovibrio bacteriovorus Tiberius	3	3
Bdellovibrio bacteriovorus SSB218315	3	3
Bdellovibrio bacteriovorus kdesi	3	2
Bdellovibrio species qaytius	3	2
Bdellovibrio species NC01	3	2
Bdellovibrio species ZAP7	3	4
Bdellovibrio reynosensis LBG001	3	3
Bdellovibrio exovorus JSS	3	1
Bdellovibrio bacteriovorus W	3	2
Bdellovibrionaceae bacterium HK-STAS-BDEL-7	3	2
Halobacteriovorax marinus SJ	3	1
Halobacteriovorax marinus BE01	3	1
Halobacteriovorax species BALOs_7	3	2
Bacteriovorax stolpii Uki2	3	1
Bacteriovorax stolpii AC01	3	1

[a] Genome GenBank accessions of species are - *Bdellovibrio bacteriovorus* HD100 - NC_005363.1, *Bdellovibrio bacteriovorus* Tiberius - NC_019567.1, *Bdellovibrio bacteriovorus* SSB218315 - CP020946.1, *Bdellovibrio bacteriovorus* kdesi CP102930, *Bdellovibrio species* qaytius - CP025734.1, *Bdellovibrio species* NC01 - CP030034.1, *Bdellovibrio species* ZAP7 - CP030082.1, *Bdellovibrio* reynosensis LBG001 - CP093442.1, *Bdellovibrio exovorus* JSS - CP003537.1, *Bdellovibrio bacteriovorus* W - CP002190.1, *Bdellovibrionaceae bacterium HK-STAS-BDEL-7* - CP060220.1, *Halobacteriovorax marinus* SJ - 016620.1, *Halobacteriovorax marinus* BE01 - CP017414.1, *Halobacteriovorax species* BALOs_7 - CP027772.1, *Bacteriovorax stolpii* Uki2 - CP025704.1, *Bacteriovorax stolpii* AC01 - CP030035.1.

Table S5: Flagellar genes clusters and their genomic organization in *Bdellovibrio* and *Halobacteriovorax*.

Genome position [a]	Flagellar genes	Gene loci [a] (corresponding flagellar gene names)											
		***Bdellovibrio bacteriovorous* HD100**											
128279-129831	2	Bd0144 (motA1)	Bd0145 (motB1)										
384778-386914	2	Bd0408 (fliC)	Bd0410 (fliC)										
494983-505184	12	Bd0530 (flgF)	Bd0531 (flgG)	Bd0532 (flgA)	Bd0534 (flgH)	Bd0535 (flgI)	Bd0536 (flgJ)	Bd0537 (flgM)	Bd0538 (flgN)	Bd0540 (flgK)	Bd0542 (flgL)	Bd0542.1 (csrA)	Bd0543 (fliW)
562130-565047	2	Bd0604 (fliC)	Bd0606 (fliC)										
570624-572439	2	Bd0610 (fliD)	Bd0611 (fliS)										
753920-754903	1	Bd0804 (fliL)											
1019726-1020232	1	Bd1076 (fliL)											
2920190-2918347	2	Bd3021 (motA2)	Bd3020 (motB2)										
2960612-2961445	1	Bd3052 (fliC)											
3172312-3170520	2	Bd3254 (motA3)	Bd3253 (motB3)										
3243522-3232783	12	Bd3329 (fliL)	Bd3328 (fliM)	Bd3327 (fliN)	Bd3326 (fliO)	Bd3325 (fliP)	Bd3324 (fliQ)	Bd3323 (fliR)	Bd3322 (flhB)	Bd3321 (flhA)	Bd3320 (flhF)	Bd3319 (flhG)	Bd3318 (fliA)
3254528-3255361	1	Bd3342 (fliC)											
3318236-3306124	12	Bd3407 (flgB)	Bd3406 (flgC)	Bd3405 (fliE)	Bd3404 (fliF)	Bd3403 (fliG)	Bd3402 (fliH)	Bd3401 (fliI)	Bd3400 (fliJ)	Bd3399 (motE)	Bd3398 (fliK)	Bd3397 (flgD)	Bd3395 (flgE)
		***Halobacteriovorax marinus* SJ**											
83792-85741	2	RS00420 (fliC)	RS00425 (fliC										
99107-97299	2	RS00485 (fliS)	RS00490 (fliD)										
176263-178208	2	RS00825 (fliC)	RS00830 (fliC)										
578687-590096	12	RS16635 (fliL)	RS02715 (fliM)	RS02720 (fliN)	RS02725 (fliO)	RS02730 (flip)	RS02735 (fliQ)	RS02740 (fliR)	RS02745 (flhB)	RS02750 (flhA)	RS02755 (flhF)	RS02760 (flhG)	RS02765 (fliA)
1257530-1257060	1	RS06170 (fliL)											
2191593-2190760	1	RS10280 (fliC)											
2701120-2699450	2	RS12855 (motA)	RS17110 (motB)										
2929527-2918133	12	RS13985 (flgB)	RS13980 (flgC)	RS13975 (fliE)	RS13970 (fliF)	RS13965 (fliG)	RS13960 (fliH)	RS13955 (fliI)	RS13950 (fliJ)	RS13945 (motE)	RS13940 (fliK)	RS17160 (flgD)	RS13925 (flgE)
3336539-3345770	12	RS16000 (flgF)	RS16005 (flgG)	RS16010 (flgA)	RS16015 (flgH)	RS16020 (flgI)	RS16025 (flgJ)	RS16030 (flgM)	RS16035 (flgN)	RS16040 (flgK)	RS16045 (flgL)	RS16050 (CsrA)	RS16055 (fliW)

[a] Positions and gene loci names are from GenBank entries NC_005363.1 and NC_016620.1 for *Bdellovibrio bacteriovorous* HD100 and *Halobacteriovorax marinus* SJ, respectively. *Halobacteriovorax marinus* SJ loci names are prefixed by 'BMS_'. *fliL* and *motA, motB* are highlighted in yellow.

Table S6: Gene expression analysis of motility, chemotaxis, cyclic di-GMP (CdG) synthesis genes and CdG effectors. Levels not connected by the same letter are significantly different by Tukey's test at 0.05% significance level.

**Gene**	**Description**	**HD100Sm AP**	**MHI154 axenic**	**MHI154 prey-dependent**	**fliL:MHI 154 prey-dependent**	**MHI154 Biofilm**	**MHI167 axenic**
bd0532 (*flgA*)	Flagellar basal-body P-ring formation protein	1 (B)	5.2 ± 0.1 (A)	5 ± 0.3 (A)	0.4 ± 0.1 (C)	NA	NA
bd0536 (*flgJ*)	Flagellar protein peptidoglycan hydrolase	1 (E)	10.1 ± 0.1 (A)	9 ± 0.0 (B)	2.9± 0.2 (D)	3.1 ± 0.2 (D)	4 ± 0.4 (C)
bd3404 (*fliF*)	Flagellar M-ring protein	1 (B)	4.1 ± 0.3 (A)	3.7 ± 0.3 (A)	1.4 ± 0.2 (B)	NA	NA
bd1075	L,D-transpeptidase	1 (B)	1.2 ± 0.1 (B)	3.1 ± 0.2 (A)	0.4 ± 0.0 (C)	NA	NA
bd3328 (*fliM*)	Flagellar motor switch protein	1 (C)	7.3 ± 0.5 (B)	15.1 ± 2.7 (A)	2.4 ± 0.1 (C)	NA	NA
bd3407 (*flgB*)	Flagellar basal-body rod protein	1 (B)	38.1 ± 5.3 (A)	6.7 ± 0.9 (B)	9.8 ± 1.3 (B)	1.2 ± 0.0 (B)	46.6 ± 6.4 (A)
bd3398 (*fliK*)	Flagellar hook-length control protein	1 (C)	8.9 ± 0.3 (A)	9.2 ± 0.7 (A)	2.3 ± 0.0 (B)	NA	NA
bd1076 (*fliL*)	Flagellar basal body-associated protein	1± 0.01 (B)	1 ± 0.0 (B)	3.4 ± 0.2 (A)	0.5 ± 0.1 (C)	0.02 ± 0.0 (D)	0.002 ± 0.0 (D)
bd0804 (*fliL*)	Flagellar basal body-associated protein	1 (C)	10.7 ± 0.8 (A)	6.7 ± 2.4 (B)	2.4 ± 0.1 (C)	3.5 ± 0.3 (B, C)	6.8 ± 1.8 (B)
bd3329 (*fliL*)	Flagellar basal body-associated protein	1 (D)	2.9 ± 0.1 (C)	26.5 ± 1.2 (A)	0.9 ± 0.1 (D)	2 ± 0.1 (C, D)	4.8 ± 0.2 (B)
bd3253 (*motA*3)	motor protein	1 (C)	8.5 ± 0.5 (A)	3.1 ± 0.6 (B, B)	1 ± 0.0 (C)	NA	NA
bd3254 (*motB*3)	motor protein	1 ± 0.1 (C)	3.6 ± 0.1 (A)	1.9 ± 0.2 (B)	0.9 ± 0.1 (C)	NA	NA

bd3021 (*motA*2)	motor protein	1 (C)	389.4 ± 14.3 (B)	11.4 ± 3.5 (C)	13.1 ± 1.2 (C)	1.4 ± 0.2 (C)	434.7 ± 14.9 (A)
bd3020 (*motB*2)	motor protein	1 (D)	288.9 ± 7.6 (B)	22.9 ± 5.2 (C)	14.3 ± 2.7 (C, D)	2.2 ± 0.2 (D)	639.4 ± 19.1 (A)
bd0144 (*motA*1)	motor protein	1 (C)	5.8 ± 0.3 (B)	13.8 ± 0.9 (A)	0.2 ± 0.0 (C)	6.5 ± 0.1 (B)	4.7 ± 1.4 (B)
bd0145 (*motB*1)	motor protein	1 (B)	15.9 ± 3.3 (D, E)	5.6 ± 2.4 (C, D)	0.7 ± 0.2 (E)	7.2 ± 0.1 (B, C)	11.5 ± 0.5 (A, B)
bd2406 (*cheA*)	Chemotaxis protein histidine kinase and related kinases	1 (C)	3.6 ± 0.1 (A)	2 ± 0.2 (B)	0.6 ± 0.1 (D)	0.02 ± 0.0 (E)	2.1 ± 0.1 (B)
bd2503 (mcp)	Methyl-accepting chemotaxis sensor/transducer	1 (C)	13.6 ± 0.3 (A)	4.5 ± 1.3 (B)	0.2 ± 0.0 (C)	1.6 ± 0.0 (C)	3.9 ± 0.6 (B)
bd0932 (mcp)	Methyl-accepting chemotaxis sensor/transducer	1 (A)	1 ± 0.1 (A)	1.1 ± 0.1 (A)	0.4 ± 0.0 (B)	NA	NA
bd1126 (mcp)	Methyl-accepting chemotaxis sensor/transducer	1 (B)	1.7 ± 0.1 (A, B)	3.5 ± 1.6 (A)	0.2 ± 0.0 (B)	NA	NA
bd0367 (*dgcA*)	Two-component response regulator with GGDEF domain	1 (D)	3.8 ± 0.2 (A)	3.6 ± 0.2 (A, B)	1.9 ± 0.1 (C)	0.4 ± 0.3 (D)	3.2 ± 0.3 (B)
bd1434 (*dgcC*)	GGDEF domain protein	1 (A)	0.6 ± 0.0 (B)	0.7 ± 0.2 (A, B)	0.8 ± 0.0 (A, B)	NA	NA
bd0742 (*dgcB*)	Sensory box/GGDEF domain protein	1 (C)	16.2 ± 1.1 (A)	2.8 ± 0.4 (B)	1.1 ± 0.2 (C)	2.1 ± 0.1 (B, C)	15.6 ± 0.6 (A)
merRNA		1 (A)	0.2 ± 0.03 (B)	0.9 ± 0.1 (A)	0.1 ± 0.01 (B, C)	0.002 ± 0.00 (C)	0.24 ± 0.02 (B)
bd3734 (*mglA*)	mutual gliding-motility protein MglA/ helps in predatory	1 (C)	14.8 ± 1.1 (A)	9.8 ± 0.2 (B)	2.7 ± 0.4 (C)	3 ± 0.2 (C)	11.5 ± 1.2 (B)

	invasion by BALO						
bd2761 (*RomR*)	essential for viability in both predatory and non-predatory modes	1 (A)	0.4 ± 0.0 (B)	NE	0.2 ± 0.0 (C)	0.2 ± 0.0 (C)	0.2 ± 0.0 (C)
bd2492 (TPR gene)	predicted tetratricopeptide repeat (TPR) domains typically involved in protein-protein interactions	1 (C)	19.7 ± 1 (A)	3.9 ± 0.8 (C)	0.6 ± 0.0 (C)	2.6 ± 0.3 (C)	10.8 ± 2.6 (B)
bd2494	TamAB-like transport activity might be required for OMP/autotranspor ter proteins involved in predation	NE	NE	NE	NE	NE	NE
bd2495	TamAB-like transport activity might be required for OMP/autotranspor ter proteins involved in predation	NE	2.9 ± 0.8 (A)	1 (B, C)	0.05 ± 0.02 (C)	0.36 ± 0.05 (C)	1.8 ± 0.4 (A, B)
bd0760	Flagellar brake protein	1 (A)	0.14 ± 0.0 (C)	0.73 ± 0.00 (B)	0.08 ± 0.00 (D)	0.001 ± 0.00 (E)	0.15 ± 0.00 (C)
bd1007	Flagellar brake protein	1 (A)	0.15 ± 0.02 (A, D)	0.58 ± 0.07 (B)	0.16 ± 0.02 (D)	0.009 ± 0.0 (B, E)	0.27 ± 0.02 (C)
Bd0081	adenylate cyclase which putatively binds to c-di-GMP	1 (B)	1.59 ± 0.18 (A)	0.24 ± 0.02 (C)	NA	NA	1.35 ± 0.14 (A)

Bd1971	cyclic-di-GMP hydrolase carries a CRP domain	1 (C)	9.46 ± 0.43 (A)	5.12 ± 1.41 (B)	NA	NA	6.61 ± 2.06 (A, B)
Bd2590	coding for a Clp-like c-di-GMP binder is cotranscribed with *bd2591*, coding for a standard cyclic AMP receptor protein	1 (A)	0.64 1.17 ± 0.04 (B)	1.17 ± 0.16 (A)	NA	NA	0.43 ± 0.03 (B)

#NA= not analysed, NE= no expression
